# Analysis of Secondary Structure Biases in Naturally Presented HLA-I Ligands

**DOI:** 10.3389/fimmu.2019.02731

**Published:** 2019-11-22

**Authors:** Marta A. S. Perez, Michal Bassani-Sternberg, George Coukos, David Gfeller, Vincent Zoete

**Affiliations:** ^1^Computer-Aided Molecular Engineering, Department of Oncology, Ludwig Institute for Cancer Research, University of Lausanne, Lausanne, Switzerland; ^2^Swiss Institute of Bioinformatics, Lausanne, Switzerland; ^3^Human Integrated Tumor Immunology Discovery Engine, Department of Oncology, Ludwig Institute for Cancer Research, University Hospital of Lausanne, Lausanne, Switzerland; ^4^Computational Cancer Biology, Department of Oncology, Ludwig Institute for Cancer Research, University of Lausanne, Lausanne, Switzerland

**Keywords:** human leukocyte antigen, HLA-I ligand presentation, computational immunology, 3D structure, heuristic search, HLA-I motif-like peptides

## Abstract

Recent clinical developments in antitumor immunotherapy involving T-cell related therapeutics have led to a renewed interest for **h**uman **l**eukocyte **a**ntigen class **I** (HLA-I) binding peptides, given their potential use as peptide vaccines. Databases of HLA-I binding peptides hold therefore information on therapeutic targets essential for understanding immunity. In this work, we use in depth and accurate HLA-I peptidomics datasets determined by mass-spectrometry (MS) and analyze properties of the HLA-I binding peptides with structure-based computational approaches. HLA-I binding peptides are studied grouping all alleles together or in allotype-specific contexts. We capitalize on the increasing number of structurally determined proteins to (1) map the 3D structure of HLA-I binding peptides into the source proteins for analyzing their secondary structure and solvent accessibility in the protein context, and (2) search for potential differences between these properties in HLA-I binding peptides and in a reference dataset of HLA-I motif-like peptides. This is performed by an *in-house* developed heuristic search that considers peptides across all the human proteome and converges to a collection of peptides that exhibit exactly the same motif as the HLA-I peptides. Our results, based on 9-mers matched to protein 3D structures, clearly show enriched sampling for HLA-I presentation of helical fragments in the source proteins. This enrichment is significant, as compared to 9-mer HLA-I motif-like peptides, and is not entirely explained by the helical propensity of the preferred residues in the HLA-I motifs. We give possible hypothesis for the secondary structure biases observed in HLA-I peptides. This contribution is of potential interest for researchers working in the field of antigen presentation and proteolysis. This knowledge refines the understanding of the rules governing antigen presentation and could be added to the parameters of the current peptide-MHC class I binding predictors to increase their antigen predictive ability.

## Introduction

The surface presentation of peptides by major histocompatibility complex (MHC) class I molecules is critical to all CD8^+^ T-cell adaptive immune responses, including those targeting tumor cells. For the majority of the peptides, the generation and loading on MHC class I molecules is a well-described antigen-processing multi-step pathway, dependent on the ubiquitin-proteasome pathway (1–4). In the first step, the ubiquitinated-proteasome (5) degrades intracellular proteins into small peptides that are released to cytosol. Peptides typically have 8–12 residues long (6), though they can range from 4 to 25 residues, depending on the organism and substrate. Afterwards, these peptides are transported into the endoplasmic reticulum (ER) by transporter associated with antigen processing (TAP) proteins. Peptides produced in the cytosol are further trimmed by peptidases, such as the endoplasmic reticulum-resident aminopeptidases ERAP1 and ERAP2, within the ER (7–10). In the end, after being transferred to the cell surface, the peptides bound to human leukocyte antigen class I (HLA-I) molecules may be recognized by CD8 T-cells. Intracellular proteins can also be cleaved in proteasome-TAP alternative pathways (11, 12), whose contribution to form HLA-I peptides may be indeed underestimated (13). Alternative pathways include the cleavage by proteases such as tripeptidyl peptidase II that can act independently or in cooperation with proteasome (11, 14), metallopeptidase insulin-degrading enzyme (15), and the intermembrane cleavage by the signal peptide peptidase (16, 17). Proteasome-TAP alternative pathways include also processes like the ER-associated degradation (18, 19) and autophagy associated vesicular pathways (20). Peptides produced in the lysozyme pathway can reach HLA-I by cross penetration (21). The present knowledge of how antigen presenting cells can self- and cross-present proteins is scarcer for integral membrane proteins when compared to solution proteins (22).

Human cells usually express three HLA-I genes, A, B, and C, but very specialized cell types can also express E, F, or G genes. HLA-A, HLA-B, and HLA-C genes are the most polymorphic of the human genome and more than 12'000 distinct alleles are documented in the human population. Humans usually have different combinations of HLA-I alleles and express up to six different HLA-I proteins (two for each one of the A, B, and C genes) (23). The majority of the HLA-I peptides are nine residues in length, but many studies have demonstrated high heterogeneity of peptide length distributions between different alleles. For example, some alleles such as HLA-B^*^51:01 show a high frequency of 8-mers, comparable to that of 9-mers, and very few longer peptides. Other alleles, such as HLA-A^*^01:01 show high frequency of peptides longer than 12-mers, which can be recognized by T-cells (24–29). Structurally, the majority of the alleles accommodate peptides with anchor residues at the second and last position. Whereas, 9-mers display a linear binding mode, longer peptides exhibit a bulge of their central portion protruding outside the HLA-I binding site. More rarely, alleles such as HLA-B^*^08:01 (30) bind 9-mers presenting anchor residues at middle positions and alleles such as HLA-B^*^57:01 and HLA-A^*^03:01 bind long peptides accommodating them with N- (31) and C- (32) terminal extensions, respectively.

Two main classes of experimental assays have been developed to identify HLA peptides: (1) *in vitro* assays [refolding assays (33), peptide-rescuing assays (34), competitive assays (35), dissociation assays (36), and surface plasmon resonance techniques (37)] and (2) mass-spectrometry (MS) based measurements (25, 38–40). Human cancer cell lines, tumors, healthy tissues and body fluids have been subject to immunopeptidomics analysis aimed at identifying cancer associated antigens among the endogenously presented HLA peptides (39, 41–49). Early MS immunopeptidomic measurements were severely limited by technical sensitivity and manual spectra interpretation. The technological progress with development of orbitrap mass analyzers and enhanced chromatographic performance led to vast improvements in mass accuracy, sensitivity, resolution, and speed (24, 39). Concomitantly, bioinformatic tools were developed to process MS data and integrate sequencing results (50, 51). This enabled the immense advancement of tumor immunopeptidomics, and the number of unique HLA-I peptides currently available from MS-based measurements is 10 times higher than 4 years ago (52). The best-established MS based measurement is based on immunoaffinity purification of HLA complexes from detergent solubilized lysates followed by extraction and purification of the peptides. The extracted peptides are then separated by high-pressure liquid chromatography and directly injected into a mass spectrometer. The resulting spectra obtained from the fragmentation of the peptides is in the end compared with *in silico* generated spectra of peptides (53). Despite great advances, MS data still suffers from some problems and several attempts are ongoing to correct them. First, only peptides that are part of the database used for spectral searches can be detected in HLA peptidomics' data, or else, the less accurate *de novo* method may be applied. Cysteine can be chemically modified by oxidation and such modifications are not included in standard MS spectra therefore identification of cysteine containing peptides is limited (25, 40). Second, peptides that are too hydrophobic or too hydrophilic might be missed applying the common purification methods that rely on retaining peptides through hydrophobic interactions with the solid phase. Some peptides might be lost because they have features that make them incompatible with ionization or lead to poor fragmentation (54). Notwithstanding the mentioned limitations, MS based methods represent the best methodology to comprehensively interrogate the repertoire of HLA peptides presented naturally *in vivo* (25, 38–40).

Recently, a large scale collection of MS-determined HLA-I (and HLA-II) binding peptides showed that sampling of peptides for HLA presentation linked to some well-determined biological processes (55). The sampling presentation of the self-proteome presented in HLA-I complexes is not random and correlates with the level of translation, expression and turnover rate (31, 39). Likewise, the cellular localization of proteins, possibly also related to the mechanism of their degradation, has an impact (55).

Pearson et al. (56) showed that the primary and secondary structure of proteins regulate the generation of HLA-I peptides. Among other findings, they have observed that source proteins, when compared to non-source, present lower hydropathy scores, greater acidic composition and a sheet conspicuous enrichment. Lower frequency of certain amino acids such as Proline in flanking regions of naturally presented HLA-I peptides has also been demonstrated (25). While binding to HLA appears to be the most important step of class I antigen presentation, the accuracy of the predictions of HLA-I peptides can be further improved by considering other factors such as protein cleavage, gene expression, source protein localization, and sequence features (25). Larsen et al. improved epitope prediction by combining binding affinity to HLA with antigen processing transport efficiency and proteasomal cleavage (57, 58). In their search for better HLA-binding predictors, Abelin et al. observed incidentally a larger representation of helices in HLA-I binding peptides than in peptides randomly chosen in the same proteins (25). This un-discussed preliminary observation is in line with the work of Bianchi et al., which found that there is an over-representation of transmembrane helices among strong HLA-I binders and therefore transmembrane helices are an overlooked source of HLA-I peptides (22).

In this work, we provide a larger-scale structural view of the HLA-I peptidomics in the source proteins. We use in-depth and accurate HLA-I peptidomics datasets, and analyze properties of the HLA-I peptides in the source proteins with structure-based computational approaches. HLA-I peptides are studied grouping all alleles together or in allotype-specific contexts. In detail, (1) we map the 3D structure of HLA-I peptides in the source proteins for which 3D structures are available in the protein data bank [PDB (59, 60)] and analyze their secondary structure and solvent accessibility, and (2) we search for differences between HLA-I peptides and several reference controls, namely reference datasets of HLA motif-like peptides. The reference datasets of motif-like peptides are created via heuristic search. The later are performed by a tailor-made algorithm able to explore the entire proteome (or just particular proteins with representation in the immunopeptidome) and converges to a collection of peptides, excluded from known HLA peptidome, which exhibit the exact same motif (matrix) as the HLA-I binding peptides. Our results clearly show that 9-mer HLA-I peptides exhibit a preference for helices in the source proteins. A comparison to HLA motif-like peptides proves that the localization bias to helical fragments in the source proteins is significant and is not entirely explained by the helical propensity of the preferred residues in the HLA-I motifs. We give possible hypothesis for the secondary structure biases observed in HLA-I peptides in the Results and Discussion section. This knowledge refines the understanding of the rules governing antigen presentation and could be added to the parameters of the current peptide-MHC class I binding predictors to increase their predictive ability.

## Methods

### HLA-I Dataset

We combined our previously published MS based HLA-I immunopeptidomics datasets into the HLA-I-MS peptide database, comprising 154'818 individual peptides purified from different human cell lines and tissues, across numerous HLA allotypes (24, 39, 40, 55, 61). The peptides length ranges from 8 to 25 amino acids.

### Mapping HLA-I Peptides Into the 3D Structure of the Source Proteins

**Figure 1:**
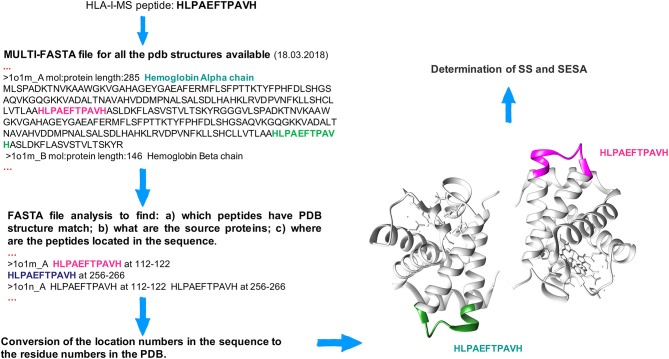
Workflow to locate peptides in the 3D structure of the source proteins. The HLA-I-MS peptide HLPAEFTPAVH is taken as example and it was matched (among others) twice in the hemoglobin alpha chain, PDBid 1O1M.

The HLA-I-MS peptides were located in the 3D structures of the source proteins, using a Perl script developed *in house*. The latter locates each individual sequence taken from the HLA-I-MS peptide database one by one in a multi-FASTA file compiling all human protein sequences for which an experimental 3D structure exists in the Protein Data Bank (www.rcsb.org) (59, 60). 52'352 Homo sapiens PDB structures were considered as of March 18, 2018. HLA-I-MS peptides from proteins/regions of proteins with unknown three-dimensional structure were not mapped (62). 41,204 individual HLA-I-MS peptides were effectively mapped on 32,883 PDB structures. The latter were downloaded from PDB and standardized. For structures with residues on alternate conformations, only the first geometry was taken. For NMR structures with several models, only the first model was used. Large structures only available in TAR archives and structures with resolution higher than 6 Å were withdrawn for technical reasons. These 168 structures represent 0.5% of the total number of structures. Therefore, their contribution is expected to be negligible. We note that one single peptide can be located multiple times within the same 3D structure and/or in different structures with high sequence similarity and/or in dissimilar proteins, as seen in the example of the HLAPAEFTPAVH peptide in hemoglobin alpha-chain: PDBid 1O1M (Figure 1). After location in the source proteins, all matched HLA-I-MS peptides were subjected to secondary structure (SS) and solvent accessibility analysis. To prevent a possible overrepresentation of certain peptides in the 3D structures, data were normalized per individual peptide, i.e., for each individual peptide with several PDB matches the measured properties were averaged over the number of matches. HLA-I-MS peptides with PDB representation are a representative subset of the entire database with nearly equal peptide length distribution, amino acid frequencies and motifs per allele, as it will be shown throughout the results section.

### Secondary Structure and Solvent Accessibility Calculation

The secondary structure (SS) and solvent accessibility of the mapped HLA-I-MS peptide sequences, were calculated using the UCSF chimera (40) package. The general scheme used in our approach was (1) to loop through all the PDB files to calculate SS and solvent accessibility for each amino acid of each protein, (2) to list SS assignments and solvent accessibility values per amino acid per PDB file, (3) to gather SS and solvent accessibility per matched peptide and ultimately (4) to average SS and solvent accessibility per HLA-I-MS peptide if several locations could be found.

SS assignments are described in HELIX and SHEET records in the PDB format. However, when the assignments were missing we invoked KSDSSP, an implementation of **K**absch and **S**ander algorithm for **d**efining the **s**econdary **s**tructure of **p**roteins (63). KSDSSP generated helix and sheet assignments by reading the position of the backbone atoms N, Cα, C, O, and the amide hydrogen. When the amide hydrogen was missing in the structure, its position was determined by KSDSSP that placed it 1.01 Å from N along the bisector of (I) the vector opposite the bisector of C-N-CA, and (II) the vector opposite the C-O vector from the previous amino acid. The best two H-bonds for each atom were then used to determine the most likely class of secondary structure for each residue in the protein. Each candidate hydrogen bond interaction was estimated and classified as hydrogen bond if the energy was at least as favorable as −0.5 kcal/mol. Helices and strands are at least three residues long. SS calculations relied only on the backbone positions. This means that a protein 3D structure with complete side chains is not mandatory. SS was proficiently determined for 41,204 structures. Peptide residues can be in alpha-helix (H), 3_10_ –helix (G), helix-5 (I), beta-bridge (B), extended strand (E), turn (T), bend (S), and coil (C). For simplicity, we organize the SS in three main groups: helix (that comprises H, G, and I), strand (that comprises E and B), and coil (that comprises T, S, and C).

Residues solvent accessibility was defined as relative solvent excluded surface area (SESA) computed with the MSMS package as implemented in Sanner MF and Olson AJ (64). Chimera calculates solvent-excluded molecular surfaces composed of probe contact, toroidal, and reentrant surface, which differ from solvent-accessible surfaces that are traced out by a probe center. SESA was computed per residue in each PDB using MSMS, with Chimera default radii for atoms and surface probe. The relative SESA were calculated by normalizing the surface area of the peptide of interest in its protein of origin, by the surface area of the same isolated peptide in a reference state, as

(1) $$\begin{array}{l} SESA=\frac{\sum Residue.area.SES}{\sum Residue.area.SES.gxg} \end{array}$$

where *Residue.area.SES* corresponds to the surface area of the individual residue in the protein of origin and *Residue.area.SES.gxg* corresponds to the surface area values per residue in a GLY-X-GLY tri-peptide, where X is the residue of interest. *Residue.area.SES.gxg* were calculated with UCSF chimera as described in Bendell (65). Peptides mapped in proteins where residues have truncated side chains were removed from our analysis since they did not allow an accurate estimation of SESA. We successfully calculated solvent exposure for 34,778 peptides of the HLA-I-MS database.

Fraction of coil/helix/strand and solvent accessibility were individually computed not only for each HLA-I-MS peptide with PDB representation as described above, but also for all human PDB structures and for all 9-mer peptides that could be found in human PDB structures (sliding windows of nine residues across all the human proteome with available 3D structure). The fraction of coil/helix/strand is computed as the average number of residues in coil/helix/strand divided by the total number of residues.

### Comparison With IEDB

To rule out a possible bias for a given SS/SESA distribution within the HLA-I-MS database, we extended our analysis to HLA-I peptides existing in the free epitope database (http://www.iedb.org) (66). 224,289 peptides restricted to HLA-I and prevenient from all assays were retrieved (HLA-I-IE). From these peptides, 69,218 HLA-I-IE peptides were mapped on PDB structures, of which 28,992 are also present on HLA-I-MS peptides set. SS and SESA were determined for HLA-I-IE peptides, including and excluding the MS determined peptides from the set, as previously described for HLA-I-MS.

Fractions of coil, helix, strand were also determined for HLA-I peptides in IEDB with half maximal inhibitory concentration (IC_50_) <500 nM (strong binders). The strong binders were taken from http://tools.iedb.org/main/datasets/, a dataset frequently used to train binding affinity predictors.

To understand if the SS bias we observe for HLA-I binding peptides also holds for HLA-II, we additionally analyzed SS for HLA-II peptides from IEDB. 89'175 peptides restricted to HLA-II and prevenient from all assays were retrieved (HLA-II-IE). From these peptides, 24,998 HLA-II-IE peptides were mapped on PDB structures (HLA-II-IE-PDB). SS were determined for HLA-II-IE-PDB peptides.

### Amino Acid Frequencies

For comparative purposes, amino acid frequencies were computed for (a) human PDB structures used in this study, including only one single PDB structure per UniProt identifier (67)–the one with best resolution–to avoid overrepresentations of certain protein families and therefore of certain amino acids, (b) HLA-I-MS and HLA-I-MS with PDB representation, (c) HLA-I-IE and HLA-I-IE with PDB representation, and (d) all human proteins listed in UniProtKB, Human-UniProt (67). Amino acid frequencies were obtained by computing the number of occurrences of a given amino acid and dividing by the total number of amino acids in the respective set.

### Searching for Bias Between HLA-I Peptides and Motif-Like Peptides

**Figure 2:**
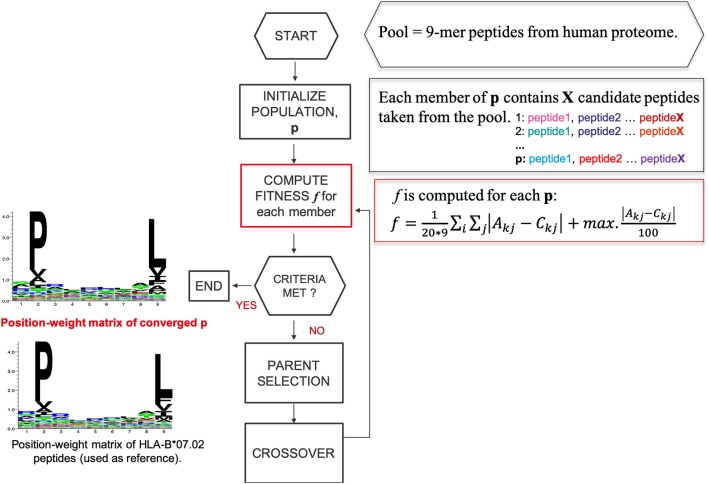
A representation of the designed heuristic search to converge to a set of motif-like peptides that exhibit the same matrix as the HLA-I binding peptides. Here HLA-B^*^07:02 is taken as example.

We designed a heuristic algorithm that searches for 9-mer peptides across the human proteome with available 3D structures and converges to a collection of peptides, called HLA-I motif-like peptides, which exhibit exactly the same motif (matrix) as the 9-mer HLA-I-MS peptides for a given allele. A representation of the designed heuristic algorithm is present in Figure 2. We have chosen length 9 since it is the dominant length in the dataset. HLA-I motif-like peptides do not include HLA-I-MS and HLA-I-IE peptides, ensuring that the motif-like peptides are not known HLA-I ligands (or not experimentally detected yet).

#### Quantification of the Distance Between HLA-I-MS Peptides and HLA-I-MS Peptides With PDB Match

Our reference sets correspond to 5 individual groups of pre-aligned 9-mer HLA-I-MS peptides that are known to bind HLA-A^*^01:01, HLA-A^*^02:01, HLA-A^*^03:01, HLA-B^*^07:02, and HLA-B^*^08:01 as determined in (40).

For each of the five groups, the 9-mer ligands were characterized using a position weight matrix, PWM_allele_ (PWM_A01:01_, PWM_A02:01_, PWM_A03:01_, PWM_B07:02_, and PWM_B08:02_), that exhibits the frequency with which each amino acid is observed at each position. Formally, given a set of *N* aligned sequences *X* of length 9, the entries of the PWM_allele_ are calculated as

(2) $$\begin{array}{l} A_{kj}=\frac{1}{N}\overset{N}{\underset{i=1}{\sum }}I\left(X_{ij}=k\right) \end{array}$$

where *i* ϵ(1, …, *N*), *j* ϵ(1, …, *9*), *k* is the set of amino acid symbols, *I(X*_*ij*_ = *k)* is 1 if *X*_*ij*_ = *k* and 0 otherwise.

Distinct PWM_allele_ are observed for each allele. Shannon sequence logos representing the motifs were generated with seq2logo using the clustering method Hobohm1, 0.63 as threshold for clustering and information content in bits (66).

PWM_allele_ were recalculated considering only the peptides with PDB match. We labeled these matrices as PWM_PDB−allele_ (PWM_PDB−A01:01_, PWM_PDB−A02:01_, PWM_PDB−A03:01_, PWM_PDB−A07:02_, and PWM_PDB−A08:02_) and their elements as B. PWM_allele_ and PWM_PDB−allele_ were compared by a function *d* that measures the matrices distances d(PWM_allele_, PWM_PDB−allele_) averaged over the number of entries (20^*^9):

(3) $$\begin{array}{l} d=\frac{1}{20^{*}9}\underset{k}{\sum }\underset{j}{\sum }|A_{kj}-B_{kj}| \end{array}$$

A small value of *d* indicates that, for the corresponding allele, the HLA-I peptides with PDB match constitute a relevant subset of HLA-I-MS, with similar amino-acid preferences for the different residue positions.

#### Creation of Reference Sets of Motif-Like Peptides

For each HLA-I allele, the heuristic search for motif-like peptides (Figure 2) consists in the following steps:

1. A pool of 597,995 individual 9-mer peptides, constructed from sliding windows of nine residues from all the human proteins with existing experimental structure in the PDB, and excluding all peptides present in HLA-I-MS and HLA-I-IE, is used as a set of possible candidates. These peptides are ranked based on a score obtained by summing the relevant probabilities at each position in PWM_allele_ **A**. Considering a sequence S = (α_1_,…, α_9_) the conformity score *cf* will be given by
  $cf=\overset{9}{\underset{j=1}{\sum }}A_{\alpha j}$, where α is the amino acid in the sequence of the peptide and *j* ϵ (1,…, *9*).
  To accelerate the convergence, a sub-pool with 10,000 peptides is constructed, containing the 6,000 top-scored peptides together with 4,000 peptides randomly taken from lower scores. Subsequent populations were constructed by selecting elements of this sub-pool. Obviously, the higher the conformity score *cf* , the more chances the peptide has to be a member of the final optimized population. However, peptides with lower scores are also needed to construct sets of motif-like peptides that reproduce exactly the PWM_allele_ matrix.
2. The initial population size is 100 and each population member (p) contains X peptide candidates randomly taken from the pre-selected pool. X is equal to the number of peptides that the reference allele contains in HLA-I-MS with PDB-match, to guarantee that we are comparing samples of the same size. The population size was adjusted to 100, a rational value considering that the sample space explored is restricted to a pre-selection of the pool.
3. PWM is calculated for each p (PWM_allele−motif−like_) and afterwards compared with PWM_allele_ via a scoring function, *f* :
  (4) $$\begin{array}{l} f=\frac{1}{20^{*}9}\underset{k}{\sum }\underset{j}{\sum }|A_{kj}-C_{kj}|+\max.\frac{\left|A_{kj}-C_{kj}\right|}{100} \end{array}$$
  Here *A*_*kj*_ is the frequency of the amino acid k in position *j* in the reference matrix and *C*_*kj*_ is the frequency of the amino acid *k* in position *j* in the matrix of *p*.
  The left-hand term of score function *f* represents the average of the module of the distance between the position weight matrices. The right-hand term characterizes the module of the maximum deviation possible between A_kj_-C_kj_. This term avoids under- and over- representation of a certain amino acid in each individual position, compared to the reference matrix, while the left-hand term ensures a global similarity between the two matrices.
  Initially, we worked with *d* as a fitness function in our heuristic search but under- and over-representation of certain amino acids at a given position were observed in the converged sets. To escape this problem, we introduced an extra term in *f* that includes the module of the maximum possible deviation between the matrices. Use of *f* led to improved convergence and accuracy in our search.
4. Population sets and their fitness values are stored.
5. The best 4% of the previous generation (4% of the members with lowest *f* ) are transferred to the next generation to guarantee convergence through elitism.
6. Crossover operations are applied to the population members between generations: the best 40% members of the previous generation are crossed over with a rate of 60–80% with members of randomly chosen parents. If the created child contains duplicate peptides, the latter are eliminated and replaced by new peptides randomly taken fom the parents and submitted to crossover.
7. This procedure loops iteratively until convergence to optimal combination of peptides. We reach convergence when the value of *f* between PWM_allele_ and PWM_allele−motif−like_ is of the same order than the *f* value between PWM_allele_ and PWM_PDB−allele_, which is considered an acceptable deviation. Therefore, independently of the allele under study, we reach convergence if *f* is lower than 0.9.
  Best probabilistic values for selection and crossover were benchmarked and are presented in Supplementary Data Sheet 2.
  The 3D structures of the converged motif-like peptides were mapped from their source protein, and SS and SESA were calculated as previously described for HLA-I-MS. Fractions of coil/helix/strand and solvent accessibility were compared with those of HLA-I-MS. All the converged motif-like sets that exhibit similar amino acid background distribution are present in SI for all the 5 groups (Supplementary Tables 1–5) and the best set, with the lowest *f* value, is presented and discussed in the main manuscript. Motif-like sets that do not present the same distribution of the *cf* conformity score as the reference set were discarded. Probability distribution is described by the fitting of a Gaussian function to the histograms of the score *cf* of the peptides in the set. See Supplementary Data Sheet 3 for accepted and rejected sets. We also analyzed adjacent residues in peptides and motif-like peptides to study if high order effects could justify the differences observed in terms of secondary structure. Statistical analyses show that adjacent residues (dipeptides) in motif-like peptides are comparable to adjacent residues in the HLA-I peptides and therefore the differences do not come from this effect. See Supplementary Data Sheet 6.

We've searched for HLA-I motif-like peptides across all human proteins. Nevertheless, not all the human proteins are presented in the immunopeptidome. Therefore, we also performed the analysis of HLA-I motif-like peptides using only the 35,598 proteins with representation on MS database (55), and excluding all peptides present in HLA-I-MS and HLA-I-IE.

For two alleles studied, HLA-B^*^44:02 and HLA-C^*^07:02, the *f* values between PWM_allele_ and PWM_allele−motif−like_ and between PWM_allele_ and PWM_PDB−allele_ are higher than 0.9 and therefore we did not reach convergence. The data for these two alleles is presented and discussed in Supplementary Data Sheet 7.

## Results and Discussion

In this section, we consider HLA-I peptides as local fragments in the 3D structure of the source proteins and determine their SS in them. Afterwards, we compare SS in HLA-I with different reference controls: (a) we investigate whether there is an enrichment in SS in HLA-I-MS-PDB peptides when compared with human PDB structures; (b) we also analyze the distribution of SS elements on a per peptide basis, and compared the results obtained for HLA-I-MS-PDB and PDB; (c) we analyze the AA composition in HLA-I peptides and PDB and; (d) we search for bias between SS in HLA-I peptides and HLA-I motif-like peptides, considering that they have the same AA composition. Finally, we give possible explanations for the biases in SS observed in HLA-I binding peptides.

### HLA-I Peptides in the Source Proteins

**Figure 3:**
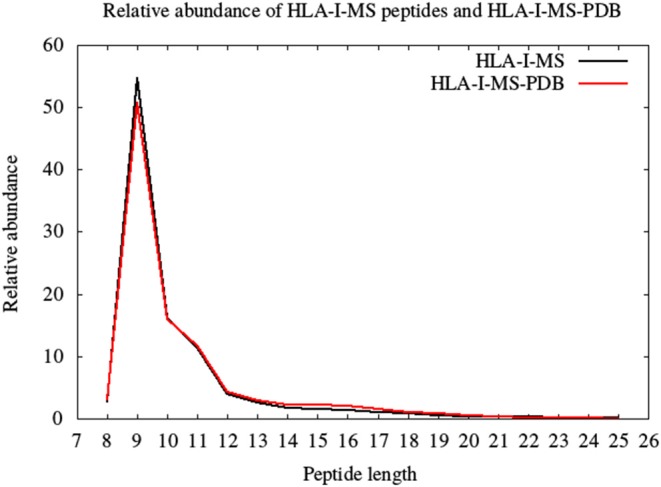
The length distribution of the HLA-I-MS peptides, in black, and of the HLA-I-MS-PDB peptides, in red. In the x-axis peptide length and in the y-axis relative abundance in %.

HLA-I-MS represents an in-depth repertoire of peptides that are naturally displayed by HLA-I molecules and cover many HLA allotypes. HLA-I-MS holds 154,818 unique peptides of which 41,204 are found in at least one experimentally determined 3D structure of a human protein, available in the PDB. We call this set of 41,204 peptides HLA-I-MS-PDB. The peptides in HLA-I-MS range from 8 to 25 amino acids long. The most frequent length is 9 with a relative abundance of 55%. It is followed by lengths 10 and 11 with relative abundances of 16 and 12%, respectively. The relative abundance per length of the HLA-I-MS-PDB peptides is nearly identical to HLA-I-MS as can be seen in Figure 3.

**Figure 4:**
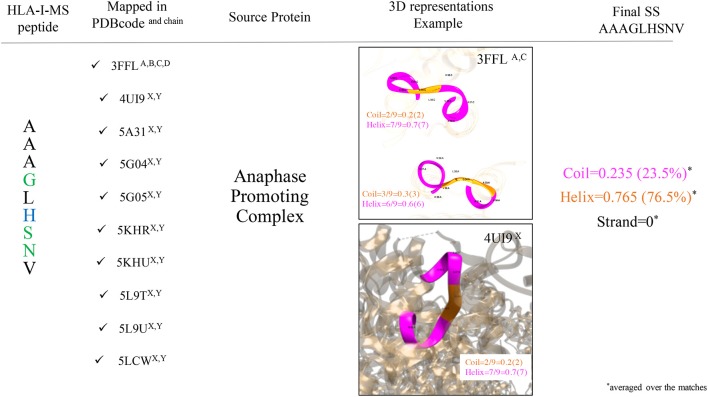
Workflow used to determine SS of the HLA-I-MS-PDB peptides, taking AAAGLHSNV as an example. AAAGLHSNV is located on chains A, B, C, and D of the structure with PDBid 3FFL and on chains X and Y of the structures with PDBid 4UI9, 5A31, 5G04, 5G05, 5KHR, 5KHU, 5L9T, 5L9U, and 5LCW. Fragments are pictured in ribbon with coil residues in orange and helical residues in magenta. AAAGLHSNV exhibits 23.5% of residues in coil and 76.5% of the residues in helix and 0% of the residues in strand.

Secondary structure elements (SS) in the source proteins were determined with KSDSSP for each HLA-I-MS-PDB peptide as described in the methods section. Figure 4 summarizes the SS determination for the HLA-I-MS-PDB peptide AAAGLHSNV, taken as an example. AAAGLHSNV can be found in 10 PDB structures with PDB*id* 3FFL, 4UI9, 5A31, 5G04, 5G05, 5KHR, 5KHU, 5L9T, 5L9U, and 5LCW. The source protein of AAAGLHSNV is the anaphase-promoting complexes or cyclosome (APC/C), a cell cycle-regulated E3 ubiquitin ligase that controls progression through mitosis and the G1 phase of the cell cycle (68). AAAGLHSNV is located on chains A, B, C, and D of 3FFL and on chains X and Y of 4UI9, 5A31, 5G04, 5G05, 5KHR, 5KHU, 5L9T, 5L9U, and 5LCW. The peptide locations on 5L9T and 5L9U were overlooked due to SS assignments failure caused by the low resolution of the structures, 6.4 Å. 3FFL corresponds to the structure of the N-terminal domain of anaphase promoting complex subunit 7 and analyzing AAAGLHSNV as a local fragment in chains A (residues 31–39) and C (residues 31–39) we can observe that in chain A, the peptide presents three residues in coil and six residues in helix, while in chain C, the peptide presents two residues in coil and seven residues in helix (see Figure 4). The secondary structure of AAAGLHSNV is normalized by averaging SS over all the matches in all the structures, signifying 23.5% of the residues in coil, 76.5% in helix and 0% in strand.

### SS: Bias Between HLA-I Peptides and Human Proteome

**Figure 5:**
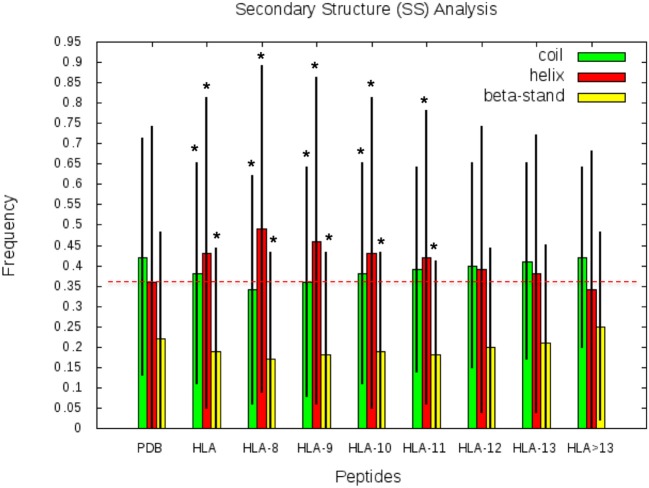
Frequency of coil, helix and strand in all human proteins with experimental structures (PDB), in HLA-I-MS-PDB taken globally (called HLA in this graph), or grouped per length (from 9- to 13-mer and > 13-mer). In the histogram, the bars are in green for coil, red for helix and yellow for strand. Levels of significance were determined by a two-tailed student's *t*-test and samples with *p* < 0.0001 are highlighted with an *, showing that the null hypotheses must be rejected and assuring that the values are significantly different from the PDB set. The black lines correspond to the standard deviation of the measure. $\text{Frequency of coil}/\text{helix}/\text{strand}=\frac{\sum residues\;in\;coil/helix/strand}{\sum residues\;in\;the\;reference\;set}$.

SS frequencies are analyzed for the HLA-I-MS-PDB dataset, taken globally or separated by peptide length, as well as for all human proteins with experimental structures in the PDB. Results are summarized in Figure 5. For simplicity, from here on, PDB refers to the human PDB structures used. In Figure 5, we can observe that PDB contain 42% of the residues in coil, 36% in helix, and 22% in strand. These values are in concordance with previous studies on the SS profile of the human proteins (69). Differently, the HLA-I-MS-PDB dataset, contains 38% of the residues in coil, 43% in helix and 19% in strand, showing a significant increase in helix of 7% (*p* <0.0001) and a decrease in coil and strand of 4% (*p* <0.0001) and 3% (*p* <0.0001), respectively. When analyzing SS variations in HLA-I-MS-PDB, we observe that the amount of helix decreases and reversely the amount of coil and strand increase when the peptide length increases (29). For 13-mer peptides the amount of helix decreases to 37%, just 1% higher than the amount of helix in PDB. For peptides longer than 13 amino acids the amount of helix becomes smaller than in the PDB. It should be noted, however, that these peptides represent <5% of the overall peptides in the database. Moreover, for peptides longer than 13 amino acids, the amount of helix decreases at a cost of an increase in strand. The ratio of structured/unstructured residues is thus equivalent to that of the PDB. The enrichment in helix is significant considering all the HLA-I-MS-PDB dataset and for HLA-I-MS-PDB peptides of length of 8, 9, 10, 11, 12, and 13 taken separately. This bias is particularly pronounced for 9-mer HLA-I-MS-PDB peptides, which represents the majority of the peptides in the database. 9-mer HLA-I-MS peptides contain 46% of the residues in helix, 8% higher than in PDB.

### SS: Bias Between HLA-I Peptides and HLA-II Peptides

SS frequencies are analyzed for the HLA-II-IE-PDB dataset, taken globally or separated by peptide length. HLA-II-IE-PDB dataset, contains 41% of the residues in coil, 33% in helix, and 26% in strand, showing a decrease in helix of 3% when compared to PDB and a significant decrease of 10% in helix when compared to HLA-I-MS-PDB. In parallel, we observed an increase in coil of 4 and 7% when compared PDB and HLA-I-MS-PDB, respectively. When analyzing SS variations in HLA-II-IE-PDB, we observed that the amount of coil is higher than 40% for all the length ranges studied, fluctuating around 40–43% for peptide lenghts between 8 and 18 mer and being higher than 43% for peptides 18 mers and higher. The highest relative frequency of peptides for HLA-II is observed at 15 amino acids length and for them the amount of helix is 35% and the amount of strand is 25%. The SS profile observed for longer HLA-I peptides is in line with the profile observed for HLA-II peptides, since peptides in the latter set are longer in length.

### SS and SESA: BIAS Between 9-Mer HLA-I and 9-Mer Peptides in Human Proteome

**Figure 6:**
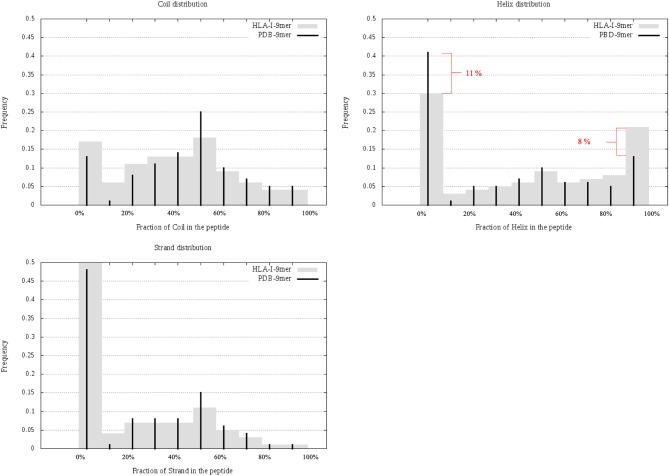
Individual graphs for helix, coil, and strand distribution as a function of the amount of the separate SS in the peptide. 9-mer HLA-I-MS in gray bars and PDB9-mer in black lines.

In this section, we considered all 9-mer peptides that are possible to construct from PDB (called PDB-9mer) and compared their SS composition to those of 9-mer peptides from HLA-I-MS-PDB. We have chosen length 9 since it is the dominant length in the dataset, representing 55% of the peptides. In Figure 6 we present individual graphs for helix, coil and strand distribution as a function of the fraction of the SS elements in the peptide. Figure 6 shows that 9-mer peptides from HLA-I-MS-PDB contain 11% less peptides with no residue in helix and 8% more peptides totally folded as helix, compared to PDB-9mer. Also, 9-mer HLA-I-MS-PDB present lower relative frequencies of peptides with 4–9 residues in coil and lower relative frequencies of peptides with 2–9 residues in strand. Regarding helix distribution, we observe that 21% of the 9-mer HLA-I peptides are fully helical (i.e., all the nine-residues are in helix in the source protein). Globally, 9-mers having at least 70% of residues in a helix in the source proteins are more frequent in HLA-I peptides than in the PDB. We conclude that not all regions of proteins are equally accessible for presentation on HLA-I and that HLA-I clearly displays more peptide residues that are in helix, notably peptides totally folded as helix in the source proteins.

Regarding solvent accessibility, we did not observe a strong preference for buried or solvent accessible peptides in HLA-I-MS-PDB when in the source proteins. The average relative SESA are similar, i.e., 47% for 9-mer PDB and 49% for 9-mer HLA-I-MS-PDB. HLA-I-MS-PDB peptides can be totally buried in the source protein, as it is the case of VFAGVFNTF mapped on 1GGT, with SESA value 0.4% or fully solvent exposed. Further details about solvent accessibility can be seen in Supplementary Data Sheet 4, in heat maps that combine solvent accessibility with fraction of secondary structure for 9-mer PDB and for 9-mer HLA-I-MS.

The results described above show a clear preference for HLA-I presentation for helical residues in their source protein. The origin of the bias could be explained by the fact that HLA-I display peptides with preferred amino-acids in specific positions (e.g., the anchor residues). Given that amino-acids have different propensities for being part of given secondary structure elements, HLA-I binding peptides, if enriched in amino acids with high helical propensities, could consequently exhibit preferred helical conformation in their source proteins. The following two sections intend to quantify the role of this scenario.

### Amino Acid Frequencies and SS Propensities: Bias Between HLA-I Peptides and Human Proteome

**Figure 7:**
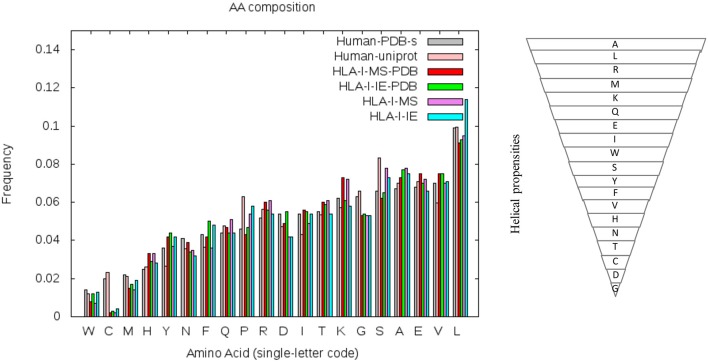
Amino acid frequencies of the human PDB structures (in gray), human proteins on UniProt (in pink), HLA-I-MS-PDB (in red), HLA-I-IE-PDB (in green), HLA-I-MS (in purple), and of the HLA-I-IE (in cyan). Amino acids in the x-axis of the graph are ordered from the lower to the higher frequencies in the PDB. Hierarchy of amino acid propensities to form helices is also shown on the right panel. All amino acids are present in the inverted pyramid except proline P, which is known to be a helix-breaker introducing a destabilizing kink in helices.

To understand if the helix enrichment in HLA-I peptides can be explained by different amino acid frequencies, we analyzed amino acid (AA) composition. To avoid potential bias coming from experiments, we analyzed AA composition not only for HLA-I-MS-PDB but also for HLA-I-IE-PDB, i.e., peptides from IEDB with PDB match. Results are shown in Figure 7, which also provides the helix propensity scale of Pace et al. (70). We observe that HLA-I-MS-PDB and HLA-I-IE-PDB exhibit different AA frequencies, but despite these differences, HLA-I-IE-PDB exhibits a SS distribution similar to HLA-I-MS-PDB: it contains 38% of the residues in coil, 42% in helix, and 20% in strand, and once again displays an enrichment in helix (6% higher when compared to PDB). The amount of helix in HLA-I-IE-PDB excluding the MS-determined peptides increases to 44% (8% higher when compared to PDB). These findings illustrate that the observed helix enrichment is database and experiment independent.

HLA-I-MS-PDB, HLA-I-IE-PDB, and PDB exhibit a strong decrease in the frequencies of proline P and serine S when compared with human proteins in UniProt (Human-UniProt), HLA-I-MS, and HLA-I-IE (see Figure 7). Proline and Serine are “disordered-promoting amino-acids.” As such they are expected to be frequently found in un-resolved, i.e., disordered, regions of the experimental structures, and consequently partially excluded from the PDB analysis. We could therefore argue that HLA-I peptides in disordered regions are underrepresented in HLA-I-MS-PDB and in HLA-I-IE-PDB, potentially leading to the bias for helix peptides. Nevertheless, HLA-I-MS and HLA-IE simultaneously exhibit enrichments in amino acids with high helical propensities when compared to Human-UniProt, which could compensate the Pro/Ser effect and reverse the bias. Notably, HLA-I-IE exhibits an enrichment in leucine L and HLA-I-MS in lysine K. This analysis is inconclusive of whether there exists an enrichment of AA with high helical propensities in HLA-I peptides that can justify the helical preference in the source proteins.

To circumvent this problem, we decided to perform the analysis in an allotype specific context, using motif-like peptides. Motif-like peptides are clusters of peptides taken from PDB that were not reported to be displayed by a given HLA-I (as far as we know) although they contain exactly the same AA frequencies and display the same motif than peptides binding experimentally to the reference allele. Under these conditions, an enrichment in helix for HLA-I binding peptides, but not for motif-like peptides, would indicate that this enrichment is independent from AA propensities.

### SS and SESA: Bias Between HLA-I Peptides and HLA-I Motif-Like Peptides in Allotype Specific Context

We analyzed allele specific HLA-I-MS peptides, searching for bias between HLA-I peptides and HLA-I motif-like peptides. We used 5 individual sets of HLA-I-MS peptides that are known to bind HLA-A^*^01:01, HLA-A^*^02:01, HLA-A^*^03:01, HLA-B^*^07:02, and HLA-B^*^08:01. These alleles exhibit different amino acid coverages, with different helical propensities and are therefore meaningful references. HLA-A^*^01:01 exhibits a preference for the negatively charged residues aspartate D and glutamate E on position 3 and for tyrosine Y on position 9. HLA-A^*^02:01 exhibits a preference for apolar residues on positions 2 and 9, predominantly leucine L in both. HLA-A^*^03:01 displays a strong preference for positively charged residues on position 9, namely lysine K. HLA-B^*^07:02 presents a distinct preference for proline P, the helix-breaker, on position 2. The latter was particularly interesting, since the presence of a proline is expected to display low helix frequencies. HLA-B^*^08:01 prefers positively charged residues on position 5, a region where the previous alleles do not show a clear preference.

**Figure 8:**
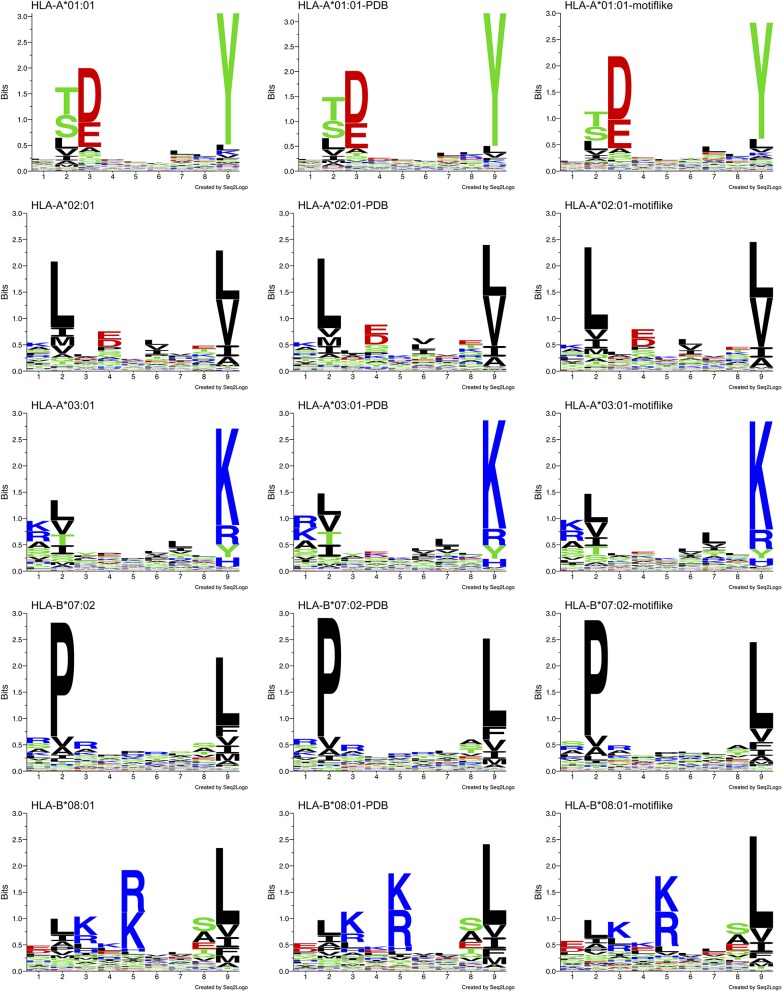
Sequence logo comparison for each of the five alleles studied: HLA-A^*^01:01, HLA-A^*^02:01, HLA-A^*^03:01, HLA-B^*^07:02, and HLA-B^*^08:01. Left: sequence logos for all known 9-mer peptide binders. Middle: sequence logos for known 9-mer peptide binders with PDB matches. Right: sequence logos for the motif-like peptides chosen by our algorithm.

Distinct PWM_allele_ are observed for each allele. Their Shannon sequence logos are shown in the left column of Figure 8. Shannon sequence logos representing the motifs with PDB match (belonging to HLA-I-MS-PDB) are shown in the middle column. Great similarities between the matrices are observed, proving that the peptides with PDB representation are a representative subset of the original dataset. The *d* values that measure the distances between PWM_allele_ and PWM_PDB−allele_ ranges from 0.61 to 0.84. The smallest *d*, 0.61, is observed for HLA-B^*^08:01 and the highest *d*, 0.84, is obtained for HLA-B^*^07:02. *f* values are ranging from 0.66 to 0.90. The lowest values are found for HLA-B^*^08:01 and HLA-A^*^03:01, and the highest value is observed for HLA-B^*^07:02. The peptides with PDB matches therefore provide subsets that accurately reproduce the motif of the reference set as can be visually inspected by the sequence logos in Figure 8, 1st and 2nd columns. The data of PWM_allele_ for each one of the alleles can be seen in Supplementary Data Sheet 1.

To answer the above questions, we decided to create populations of 9-mer peptides with PDB matches throughout the entire human proteome. These peptides are selected to show the same PWM than HLA-I-MS peptides binding a given allele, although they do not belong to HLA-I-MS themselves. Therefore, these peptides show the same residue distributions, in each peptide position, as the experimentally-determined HLA binders. As a consequence, if these peptides exhibit a lower propensity to be helical in their source protein than those belonging to HLA-I-MS, this would indicate that the increase in helical peptides in HLA-I-MS is not simply due to a higher frequency of amino acids with superior helix propensities, but that other mechanisms are playing a role.

Searching for 9-mer peptides through the entire human proteome with PDB matches implies the sampling of 597,995 sequences. The repetitive creation of subsets adding sequences one by one until reproducing the PWM_allele_ would be unfeasible. For example, for HLA-A^*^03:01, an infinite number of combinations of 989 peptides (size of the reference set) could be constructed from the ~600,000 9-mer peptides. Then, these combinations would require an individual PWM_allele−motif−like_ calculation, to be finally compared with PWM_A03:01_ via *f*. The large search space obviously prevents any systematic enumeration. Therefore, we decided to opt for a heuristic search, which is generally efficient for the search through high dimensional spaces. Here, we used an in-house developed heuristic exploration that searched for 9-mer peptides across the PDB and converged to a collection of peptides that exhibited the same matrix (PWM_allele_) as the 9-mer HLA-I-MS peptides for a given allele. HLA-I motif-like peptides do not include HLA-I-MS and HLA-I-IE, ensuring that the motif-like peptides are not known HLA-I ligands. However, we cannot exclude that they could be HLA-I ligands, but so far they were not experimentally detected (or not publicly available). Our heuristic search based on a genetic algorithm, described in the Methods, was carefully designed for this purpose. The initial population is randomly created from the pre-selection of the human proteome and each population member is defined as a set of X peptides. X is the number of peptides that the reference allele contains with PDB match to guarantee comparing samples of the same size. The number of peptides in HLA-A^*^01:01, HLA-A^*^02:01, HLA-A^*^03:01, HLA-B^*^07:02, and HLA-B^*^08:02 are 467, 846, 989, 736, and 709, respectively. A PWM was calculated for each population member. This PWM_allele−motif−like_, was compared to the experimental reference via the fitness function *f* (see Methods). Numerous trials were performed to fine-tune crossover and selection probabilities. The resulting convergence trials can be seen in Supplementary Data Sheet 2. After adjustment of these probabilities, 1,000 generations (i.e., 1,000^*^100 members evaluated) were sufficient to find near-optimal solutions. All converged motif-like sets that exhibit similar amino acid background distribution are present in SI for all five groups (Supplementary Tables 1–5) and the best set is presented and discussed in Table 1. The best motif-like sets correspond to peptides from the human proteome that converged to the lowest *f*. Generated motif-like peptides have the same amino acid background distribution, the same distribution of the conformity score *cf* and strictly follow HLA-I-MS motifs, with *f* values ranging from 0.54 to 0.79 and *d* values ranging from 0.50 to 0.73. These values are similar to the distance between PWM_allele_ and PWM_allele−PDB_, supporting the relevance of the sets of motif-like peptides. Shannon logos of the best motif-like sets are shown in the right column of Figure 8. The analysis of the fraction of coil/helix/strand/solvent accessibility of the converged motifs and comparison with structural characteristics of the respective motifs in HLA-I-MS is presented in Table 1. *d* and *f* values for HLA-I-MS peptides and motif like peptides are also present in the table. PWM_motiflike−A01:01_, PWM_motif−like−A02:01_, PWM_motiflike−A03:01_, PWM_motiflike−B07:02_, and PWM_motiflike−B08:01_ are given in Supplementary Data Sheet 1.

**Table 1:** Comparison between HLA-I-MS peptides, motif-like peptides# from PDB with representation on immunopeptidome and motif-like peptides from _PDB with representation on proteome for five different alleles: HLA-A^*^01:01, HLA-A^*^02:01, HLA-A^*^03:01, HLA-B^*^07:02, and HLA-B^*^08:01.

**Allele**		***d***	***f***	**Coil**		**Helix**		**Strand**		**SESA**	
				**AVG**	**STDEV**	**AVG**	**STDEV**	**AVG**	**STDEV**	**AVG**	**STDEV**
HLA-A^*^01:01	HLA-1-MS peptides	0.62	0.67	0.376	0.007	0.455	0.010	0.168	0.006	0.480	0.003
	motif-like peptides#	0.87	0.99	0.406^*^	0.007	0.403^*^	0.009	0.190^*^	0.006	–	–
	motif-like peptides	0.66	0.73	0.412^*^	0.006	0.393^*^	0.009	0.195^*^	0.006	0.468	0.004
HLA-A^*^02:01	HLA-1-MS peptides	0.64	0.68	0.279	0.004	0.560	0.007	0.160	0.004	0.456	0.002
	motif-like peptides#	0.63	0.69	0.300^*^	0.005	0.544^*^	0.007	0.153	0.004	–	–
	motif-like peptides	0.50	0.54	0.300^*^	0.004	0.544^*^	0.006	0.160	0.004	0.443	0.003
HLA-A^*^03:01	HLA-1-MS peptides	0.62	0.66	0.355	0.004	0.468	0.006	0.177	0.004	0.500	0.002
	motif-like peptides#	0.92	0.99	0.350	0.004	0.474	0.006	0.174	0.004	–	–
	motif-like peptides	0.73	0.79	0.353	0.005	0.461	0.005	0.186^*^	0.004	0.485	0.003
HLA-B^*^07:02	HLA-1-MS peptides	0.84	0.90	0.474	0.006	0.335	0.007	0.189	0.004	0.492	0.003
	motif-like peptides#	0.84	0.95	0.484	0.005	0.319^*^	0.006	0.196	0.004	–	–
	motif-like peptides	0.66	0.72	0.510^*^	0.006	0.288^*^	0.007	0.202^*^	0.004	0.500	0.003
HLA-B^*^08:01	HLA-1-MS peptides	0.61	0.66	0.260	0.005	0.600	0.008	0.140	0.005	0.480	0.003
	motif-like peptides#	0.75	0.85	0.271	0.005	0.572^*^	0.008	0.155	0.004	–	–
	motif-like peptides	0.62	0.67	0.290^*^	0.007	0.564^*^	0.009	0.146	0.006	0.480	0.003

*Values for the fitness functions d and f compare (I) HLA-I-MS (known binders) and HLA-I-MS-PDB (known binders with PDB match), (II) motif-like (non-binders, yet with similar PWM) and HLA-I-MS peptides. Average (AVG) of the amount of coil, helix, strand, and SESA and respective standard deviation (STDEV) is present for each allele*.

*STDEV is the standard deviation of the mean calculated considering 100 times 80% of the peptides randomly taken*.

Table 1 shows that the HLA-I-MS alleles present different ratios of coil/helix/strand in the source proteins. In this study, HLA-B^*^08:01 is the allele with the largest fraction of peptide residues in helix, 0.600 (60.0 ± 0.8%), and HLA-B^*^07:02 is the allele with the smallest amount of peptide residues in helix, 0.335 (33.5 ± 0.7%). These differences are not surprising considering that the motifs have different amino acid preferences. HLA-B^*^08:01 presents a preference for amino acids with higher helix propensities in different positions of the peptide, such as leucine in positions 2 and 9 and arginine and lysine in positions 3 and 5. HLA-B^*^07:02, on the other side, presents a strong preference for proline, a well-known helix breaker, in position 2, justifying the smaller amount of helix observed. Although these differences are not surprising, the fact that the converged motif-like peptides always present a lower number of residues in helices compared to the peptides binding the reference allele (whatever the allele) is significant. The motif-like peptides for HLA-B^*^08:01 present 56.4 ± 0.9% of residues in helix, 3.6% lower compared to the reference (*p* <0.0001). The motif-like peptides for HLA-B^*^07:02 present 28.8 ± 0.7% of residues in helix, 4% lower compared to the reference (*p* <0.0001). HLA-I-MS-PDB peptides binding to HLA-A^*^01:01 and HLA-A^*^02:01 present 45.5 ± 1.0 and 56.0 ± 0.7% of helices, respectively. These values decrease to 39.3 ± 0.9 and 54.4 ± 0.6%, respectively, in the motif-like peptides. Only HLA-A^*^03:01 present almost equivalent amounts of helix in HLA-I-MS and in the motif-like, i.e., 46.8 ± 0.6 and 46.1 ± 0.5%, respectively. Averaging over all 64 converged motif-like sets for HLA-A^*^03, Table HLA-A^*^03:01 in Supplementary Table 3, we find again a similar amount of helix (46.1 ± 0.9%). To sum up, for four alleles we see a decrease in helix in the motif-like peptides, always compensated by an increase in coil, sometimes with an increase in strand. Analysis of motif-like peptides from proteins with representation on immunopeptidome (motif-like peptides# in Table 1) shows again a significant decrease in helix for the same four for alleles but to a smaller extent. The limited decreases in the % helix for motif-like peptide# when compared to the decreases observed for motif-like peptide correlates with the fact that the PDB structures with representation in immunopeptidome have 2% more residues in helix than the PDB structures with representation on proteome. The latter observation is in line with our findings showing that HLA-binding peptides are enriched in helices in the source protein.

These results support the fact that 9-mer HLA-I binding peptides prefer helical secondary structures in their proteins of origin, and that this preference is not because there is a higher frequency of amino acids with high helical propensities in HLA-I motifs. The helical enrichment holds for 8-mers and possibly also for 10-mers, to a lower extent (Figure 5). Nevertheless, we have 18 times and 4 times less data for 8-mers and 10-mers, respectively, than for 9-mers, which prevents a thorough analysis as performed for 9-mers. In total, covering 8- to 10-mers, the helical enrichment could be observed for more than 74% of the peptidome identified experimentally in our datasets. Interestingly, the enrichment in helices is not present in long peptides, which exhibit less helical fragments compared to PDB. The enrichment in helix also does not hold for HLA-II peptides, since peptides in this set are longer in length. This result is in line with a previously published analysis (29), showing an increased frequency of glycines in long MHC-binding peptides. The latter was hypothesized to enable long peptides to adopt bulging conformations more easily.

### Possible Hypothesis for the Bias in SS Observed for HLA-I Binding Peptides

Many different proteolytic systems may generate antigenic peptides and the proteasome could be responsible for the release of the majority of them. Non-proteasomal proteolytic pathways also generate antigenic peptides and their contribution is most probably underestimated (13). It has been found that the largest frequency of proteolytic cleavages, by proteasome or other proteases, occur in coil regions (5, 71, 72). Proteasome and other proteases differ in the mode of action: while proteasome degrades proteins in highly successive manner (73), other proteases perform single cuts leaving the protein afterwards. If the source protein in the cell is cleaved following a proteasome pathway, the protein regions more prone to unfold will be ubiquitinated (5) and afterwards will suffer multiple sequential cuts, converting the protein into oligopeptides. Additional trimmings by other proteases will be done preferentially in the coil portion of the oligopeptide products, leaving more helical residues together.

If the source proteins follow a non-proteasomal pathway, they will experience independent cleavages preferentially at coil positions, leaving more helicoidal peptides to be displayed. To sum up, prior to loading on the HLA complexes, the peptides must be cleaved or trimmed in N-term and C-term to be available, but at the same time must be stable enough to survive destruction and to be displayed by HLA. The higher resistance of helices to proteolysis could explain the higher frequency of helical regions among HLA-I binding molecules. Residues adjacent to the 9-mer HLA-I peptides presented in the ligandome also exhibit an enrichment in helical residues in the source proteins. Indeed, 47% of the residues immediately before the N-term of the 9-mer and 44% of residues immediately after the C-term are helical residues in the source proteins, i.e., 11 and 8% more than the average of the PDB. This does not mean that these residues are still in helix when in oligopeptide products in the cytosol but indicates that the residues adjacent to HLA-I peptides in the source proteins are an extension of the helix which promotes the peptide stability. Figure 4 represents an example where the residues adjacent to the peptide are in helix in the source protein. We also observe a decrease in strand for 9-mer suggesting that during the processing of the peptides in the cell, more peptides that were in such secondary structures in the source proteins were broken. Peptides in helix can also be unstable. Nevertheless, the amount of helix in the human structures is roughly the double of the amount of strand and, during the processing, more helical 9-mer peptides are escaping destruction.

Additional studies could be useful to verify this hypothesis, by investigating experimentally the role of proteasome and other proteases like ERAP1 on the generation of the peptidome, following for example the work of Admon et al. (10, 49, 74). A very recent publication on ERAP1 inhibition showed that the average predicted affinity of MHC-I binding peptides was enhanced, by reducing presentation of sub-optimal long peptides and increasing presentation of many high-affinity 9–12-mers, suggesting that baseline ERAP1 activity in this cell line (A375, melanoma cells) is destructive for many potential epitopes (74). Based on the published results we hypothesize that the edited immunopeptidome in ERAP1 inhibited melanoma cells could still present a helix enrichment because: (1) ERAP1 inhibition increased the presentation of 9–12-mers peptides which were found in our study to show higher helical content when compared to longer peptides; (2) ERAP1 inhibition increased the frequency of N-terminal AA such as ALA, LEU, TYR, and MET which are all known to have a high helical propensity; (3) the inhibition does not affect the basic sequence motifs of the presented peptides. However, we cannot argue if ERAP1 individually can favor or disfavor the HLA-I presentation of peptides enriched in helices in the source proteins, as many other factors that are not related with the cleavage by this aminopeptidase are also responsible for the immunopeptidome edition. Indeed, peptide processing mechanisms via proteolysis, or at least via ERAP1, may not be the exclusive factor to produce helix enrichment.

TAP transport, for example, is an important step in antigen processing that precedes MHC binding in the conventional proteasome pathway and therefore takes a significant contribution to peptide selection. Considering that the C-terminal portion of the peptide that binds TAP prefers hydrophobic or basic residues (75) and that transmembrane helices require hydrophobic residues to span membranes, we envision an enrichment in helices in the C-terminal portion of the peptide. We also envision a preference to helices in the N-terminal portions of the peptide as proline is a helix breaker and have a deleterious effect in TAP binding affinity specially if located on position P1 and P2 (76).

Among various processes playing a role in antigen presentation, such as abundance of the precursor protein, efficiency of the cleavage, the stability of the peptide in the cytosol, the stability of the HLA-I complex and the affinity of antigen peptides, the latest one is considered to be a major determinant. When bound to HLA-I, peptides take an extended or bulged conformation. Therefore, the binding of peptides that are initially helical can be penalized from a conformational point of view. On the contrary, peptides that have a propensity to be unstructured are less penalized from this point of view, although they are more likely to be disfavored from an entropic perspective. All in all, HLA-I strong binders (IC_50_ <500 nM in IEDB) exhibit an increase in coil. Of note, these strong binders do not include MS data for which affinity is not measured. Further discussion can be seen in Supplementary Data Sheet 5. This supports that the bias we observe for helical peptides in the complete collection of HLA binders (whatever their affinity) is more likely related to HLA-I processing than to their affinity for HLA-I. The actual immunopeptidome results from a combination of processes that take place in the cell. Some of them can favor or disfavor helices, but globally, we observe that antigen processing results in an enrichment in helix in the HLA-I binding peptides in their source proteins. More knowledge in the field of antigen processing would be required to identify unambiguously the origin of this enrichment.

Our findings can now be added to the parameters of the current peptide-MHC class I binding predictors to increase their antigen predictive ability. Taking as an example NetCTL (57, 58) that identifies epitopes by combining the prediction methods for MHC-I affinity, TAP transport efficiency and C-terminal cleavage and has demonstrated that the integrative approach has a predictive performance that is superior to predictions of MHC-I affinity alone. The prediction of epitopes in NetCTL might be improved by combining the three previous approaches with a fourth approach that determines epitopes SS in the source protein. NetCTL predicts cytotoxic T cells epitopes in protein sequences (single sequences or several fasta sequences are given as starting point). Therefore, it could be possible to detemine the SS features for a given sequence, for example using the protein annotation features from UNIPROT (67, 77) or a tool that predicts secondary structure such as JPRED (78). Then, the candidate epitopes could be scored based on their SS composition, with peptides with higher helical composition scoring higher. Ultimately, the global prediction score of NetCTL could be retrained to incorporate a weighted sum of the four individual prediction scores from the four approaches. Large scale training and test sets would be needed to optimize the predictive performance including SS.

## Conclusion

Large peptide datasets are ideal for understanding how protein structure context contribute to peptide processing and presentation by HLA-I. In this study we refined our understanding of processing rules by analyzing the topology of MS-based peptides displayed by HLA-I (i.e., the HLA-I-MS) in the 3D structure of the source proteins. To account for potential biases coming from MS experiments, another dataset of HLA-I peptides taken from IEDB, excluding MS determined ones, was used afterwards. Our analyses of HLA-I peptides matched to protein 3D structures support the helix enrichment in the source proteins for 9-mer HLA-I peptides.

Our study clearly shows that 9-mer HLA-I peptides, that represent the majority of the HLA-I peptides, exhibit localization bias to helical fragments in the source proteins. One possible explanation for such an enrichment comes from the fact that prior to loading on the HLA complexes, the peptides must be cleaved or trimmed in N-term and C-term to be available, but at the same time has be stable enough to be displayed to HLA. Therefore, the higher resistance of helices to proteolysis could explain the higher frequency of helical regions among HLA-I binding molecules.

This knowledge provides new hints that refine our understanding of the rules of antigen processing and presentation. These findings could possibly be added to the parameters of the current peptide-MHC class I binding predictors to increase their antigen predictive ability.

## Data Availability Statement

Publicly available datasets were analyzed in this study. This data can be found here: www.iedb.org.

## Author Contributions

MP and VZ: conception and design of the work, analysis and interpretation of data, and manuscript writing. MB-S and GC: HLA-I peptidomics dataset and critical revision for important intellectual content. DG: HLA-I peptides divided per allele and critical revision for important intellectual content. All authors approved the final version of the manuscript and agreed to be accountable for all aspects of the work in ensuring that questions related to the accuracy or integrity of any part of the work are appropriately investigated and resolved.

### Conflict of Interest

The authors declare that the research was conducted in the absence of any commercial or financial relationships that could be construed as a potential conflict of interest.
