# Supplementary material for: Analysis of Secondary Structure Biases in Naturally Presented HLA-I Ligands
Source: Front Immunol. 2019 Nov 22;10:2731. doi: 10.3389/fimmu.2019.02731 (PMC6883762; doi:10.3389/fimmu.2019.02731)
Supplement: Supplementary file 7 [file Data_Sheet_2.PDF]

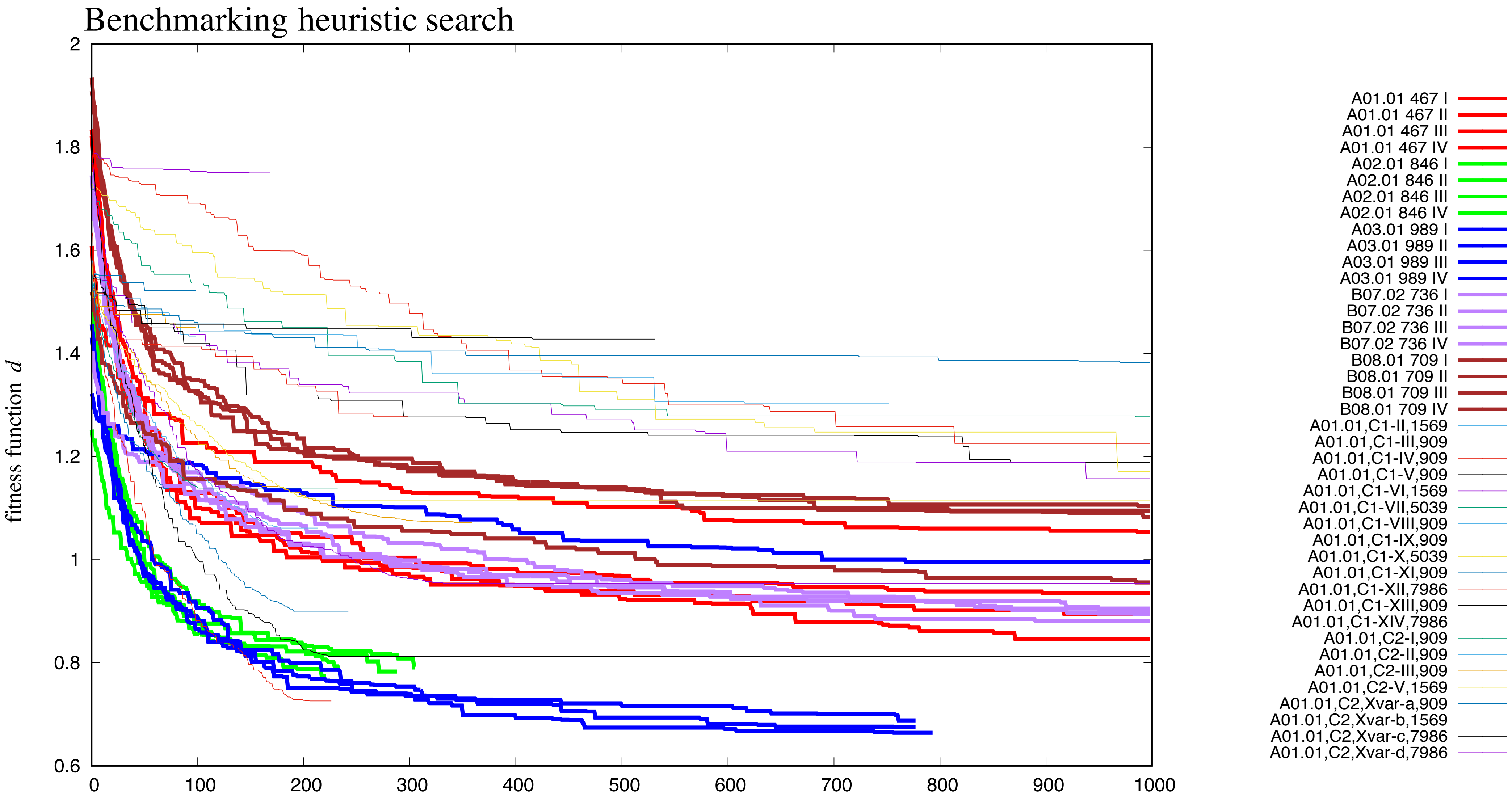

Figure. Interpreting convergence of the heuristic search. Several independent runs to search for HLA-A*01:01 motif-like peptides with different selection and crossover rates are presented with thiner lines. Optimal convergence (faster and to lower values) is obtained when 4% of the best members of the previous generation are selected to go to the next generation and 40% of the members of the previous generations are crossed over with a rate of 60% with members of randomly chosen parents. Independent runs using optimal parameters are presented with thicker lines, red for HLA-A*01:01, green for HLA-A*02:02, blue for HLA-A*03:01, violet for HLA-B*07:02 and brown for HLA-B*08:01. Within the same allele independent runs converge to different combinations of motif-like peptides – several independent runs were therefore executed after this benchmark in order to obtain the final reference sets of motif-like peptides per allele.
