# Supplementary material for: Analysis of Secondary Structure Biases in Naturally Presented HLA-I Ligands
Source: Front Immunol. 2019 Nov 22;10:2731. doi: 10.3389/fimmu.2019.02731 (PMC6883762; doi:10.3389/fimmu.2019.02731)
Supplement: Supplementary file 9 [file Data_Sheet_4.PDF]

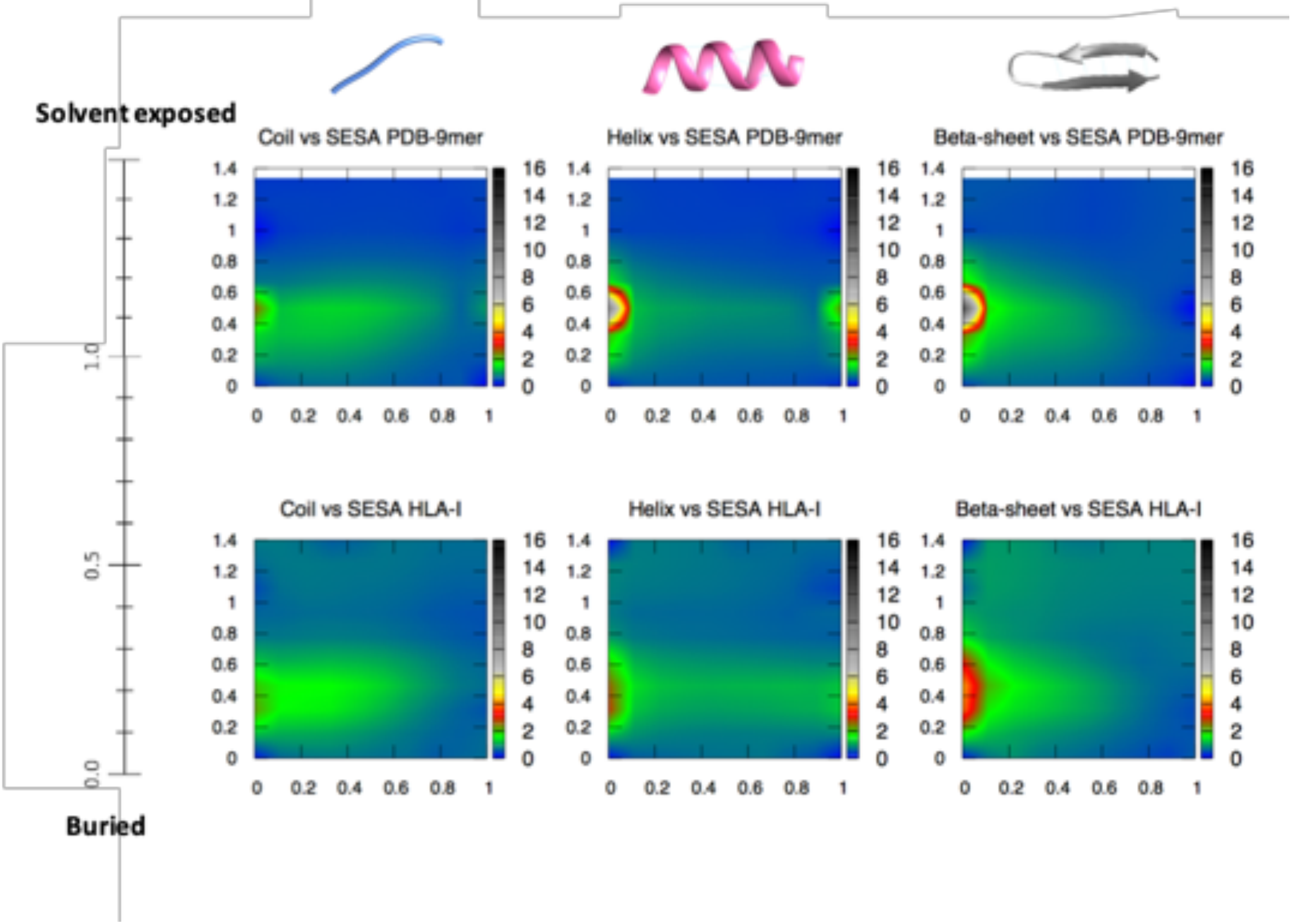

Figure. Heat-maps showing the relationship between the amount of coil/helix/sheet (X-axis) SESA (Y-axis) and in the peptide for HLA-I-MB-PDB (down row) and for 9-mer peptides possible to construct from PDB (upper row). The color code represents the relative density of points.
