# Supplementary material for: Analysis of Secondary Structure Biases in Naturally Presented HLA-I Ligands
Source: Front Immunol. 2019 Nov 22;10:2731. doi: 10.3389/fimmu.2019.02731 (PMC6883762; doi:10.3389/fimmu.2019.02731)
Supplement: Supplementary file 10 [file Data_Sheet_5.PDF]

Table SI. Average (AVG) of the amount of coil, helix, strand and respective standard deviation (STDEV) is present for HLA-I peptides with IC50<500nM (strong binders). The strong binders were taken from IEDB http://tools.iedb.org/main/datasets/, a dataset frequently used to train binding affinity. From the dataset, artificially synthesized peptides that are not processed in the cell and peptides without PDB representation were excluded.

HLA-I binders IEDB IC50 < 500nM

Coil		Helix		Strand	
AVG	STDEV	AVG	STDEV	AVG	STDEV
0.590	0.370	0.290	0.400	0.120	0.190

This table is used to validate that the enrichment in helix is related with antigen processing and not necessarily with binding affinity of the peptides to HLA-I. Any correlation between secondary structure and immunogenicity based on the helical diminishment observed for IEDB strong binders in the table might be risky. First, because not all the strong binders are immunogenic. Wang et al., for example, shows that approximately one-third of the HLA-I strong binders are non-immunogenic[1]. Second, the number of peptides with experimental binding affinities is scarce (strong binders from IEDB and with PDB match correspond to less than 1% of the overall number of peptides studied) and the binding affinities were measured in different experimental conditions, introducing biases. Third, the data used is allele independent, which means that we measure the secondary structure for the peptide if it is a strong binder at least for one allele. This introduces some errors as binding affinities are allele dependent. For example, for a large collection of ~30,000 dengue virus-derived peptides only 0.3% were predicted to bind HLA A*01:01, whereas nearly 5% were predicted to bind A*02:01 [2]. Fourth, immunogenicity depends on a plethora of effects, so that isolating the influence of peptide secondary structure in their source protein could be difficult. The abundance of HLA-I peptides is affected by the binding affinity of the peptide to HLA-I, the stability of the complex HLA-I peptide, the abundance of the precursor protein and the efficiency of HLA-I peptide processing. The recognition of the complex HLA-I peptide by TCR depends on other additional effects.

1. Wang, S., et al., *Analyzing the effect of peptide-HLA-binding ability on the immunogenicity of potential CD8+ and CD4+ T cell epitopes in a large dataset.* Immunol Res, 2016. **64**(4): p. 908-18.
2. Paul, S., et al., *HLA class I alleles are associated with peptide-binding repertoires of different size, affinity, and immunogenicity.* J Immunol, 2013. **191**(12): p. 5831-9.
