# Supplementary material for: Analysis of Secondary Structure Biases in Naturally Presented HLA-I Ligands
Source: Front Immunol. 2019 Nov 22;10:2731. doi: 10.3389/fimmu.2019.02731 (PMC6883762; doi:10.3389/fimmu.2019.02731)
Supplement: Supplementary file 11 [file Data_Sheet_6.PDF]

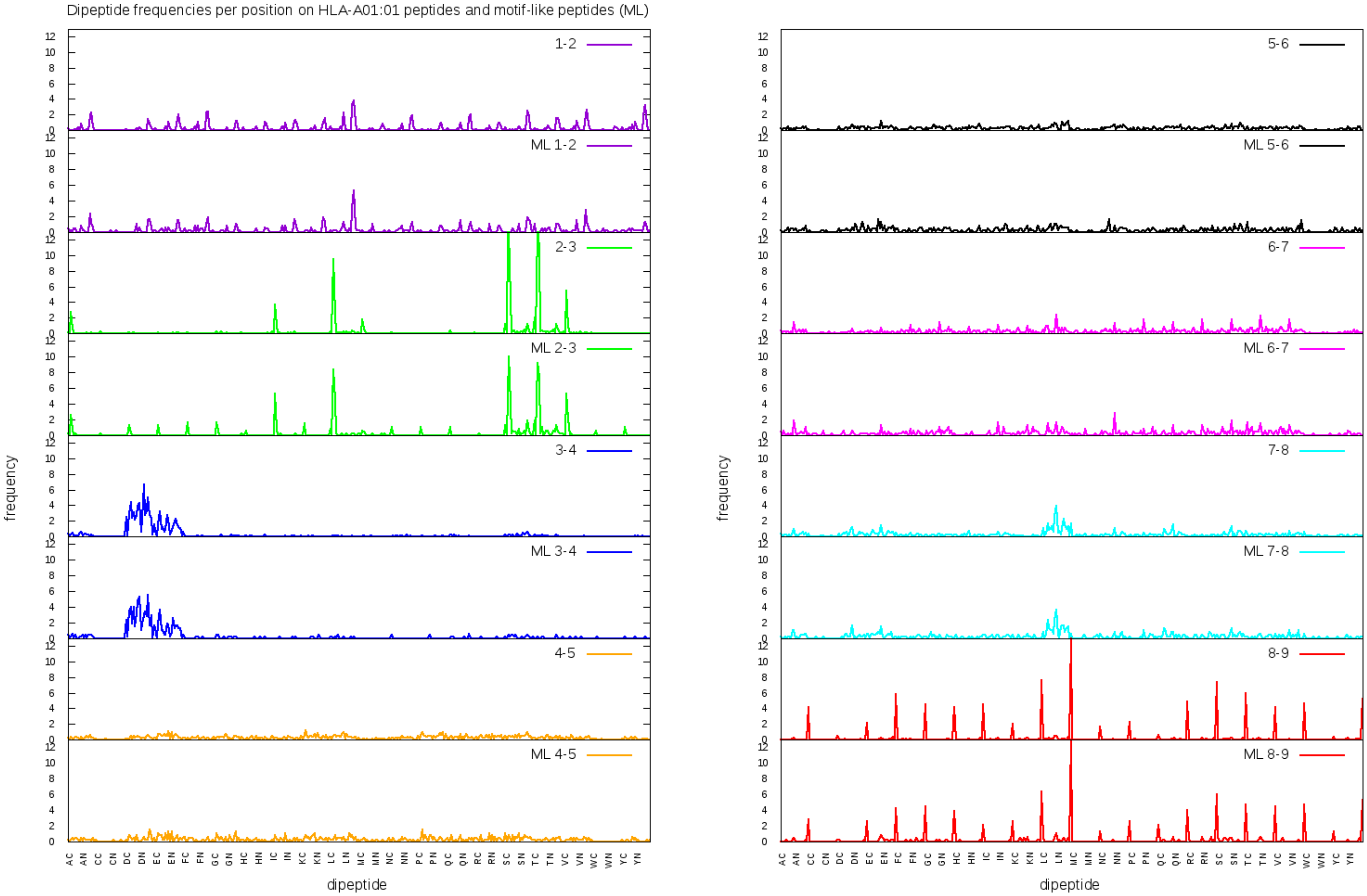

Figure. Dipeptide frequencies per pair-positions (1-2, 2-3, 3-4, 4-5, 5-6, 6-7, 7-8, 8-9) on 9-mer HLA-A*01:01 peptides and on 9-mer HLA-A*01:01 motif-like peptides (ML). 400 pairs of amino acids are possible to construct and therefore represented on x-axis. Just a few amino acids pairs are labeled for simplicity.

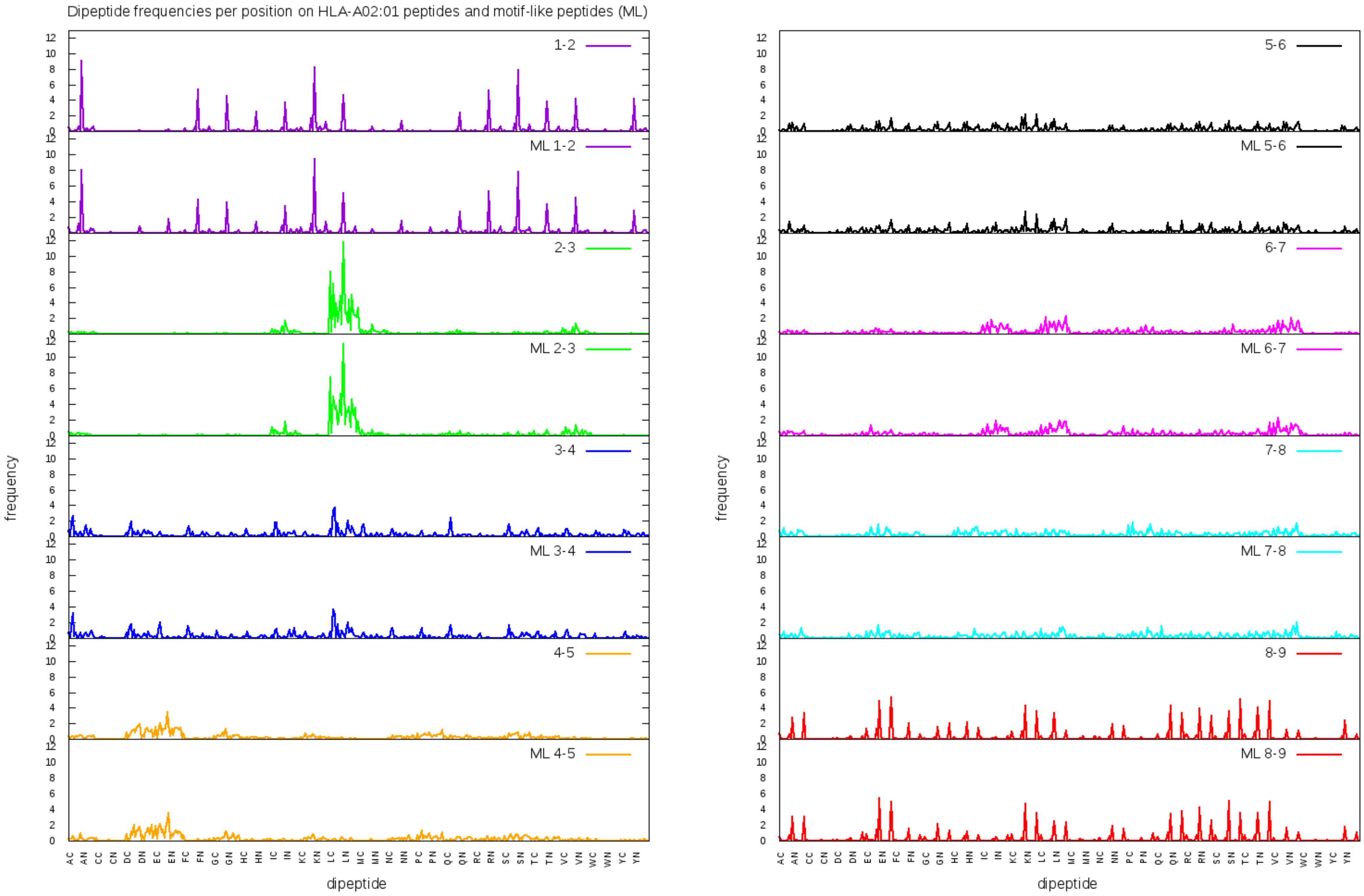

Figure. Dipeptide frequencies per pair-positions (1-2, 2-3, 3-4, 4-5, 5-6, 6-7, 7-8, 8-9) on 9-mer HLA-A*02:01 peptides and on 9-mer HLA-A*02:01 motif-like peptides (ML). 400 pairs of amino acids are possible to construct and therefore represented on x-axis. Just a few amino acids pairs are labeled for simplicity.

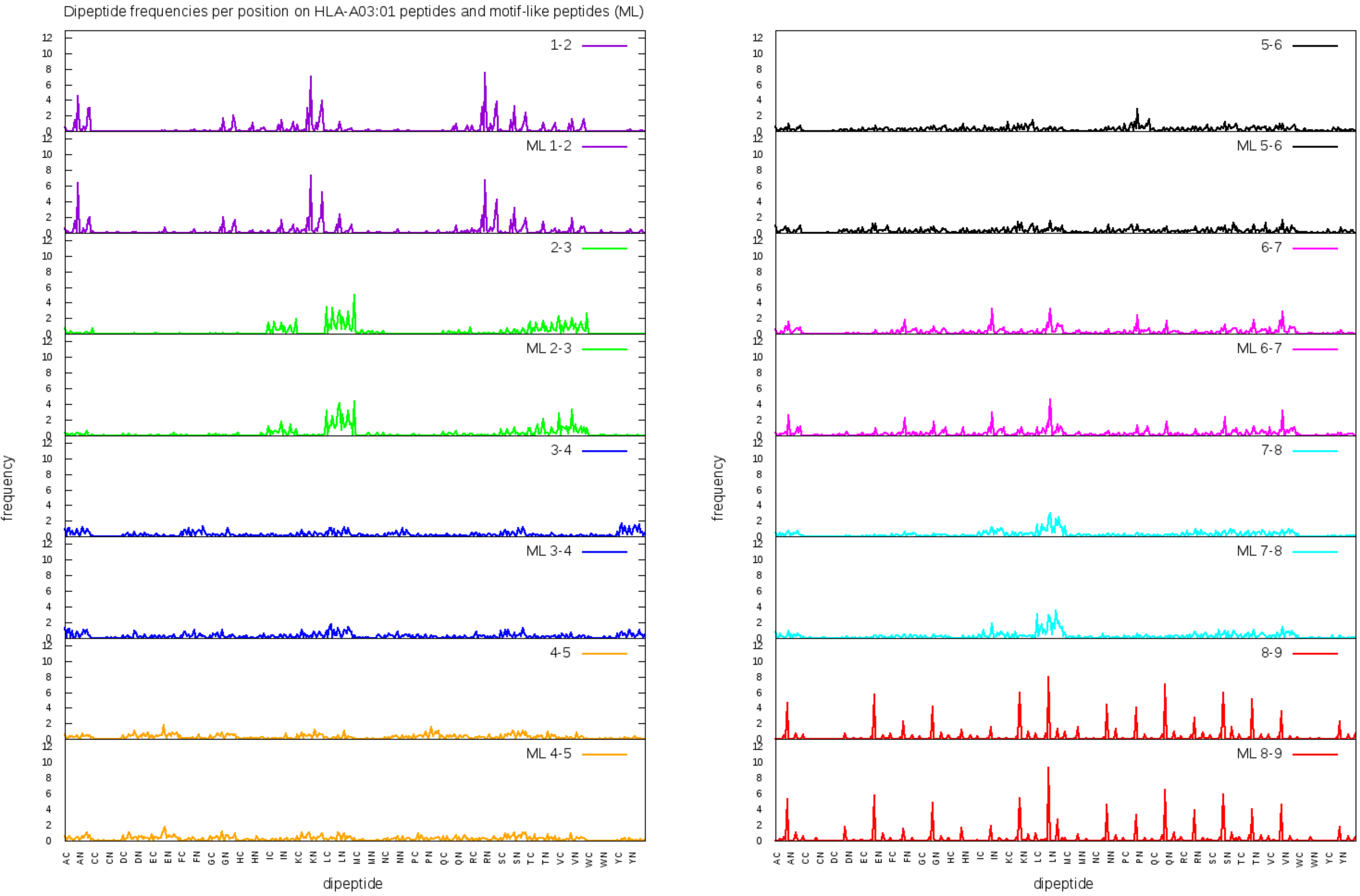

Figure. Dipeptide frequencies per pair-positions (1-2, 2-3, 3-4, 4-5, 5-6, 6-7, 7-8, 8-9) on 9-mer HLA-A*03:01 peptides and on 9-mer HLA-A*03:01 motif-like peptides (ML). 400 pairs of amino acids are possible to construct and therefore represented on x-axis. Just a few amino acids pairs are labeled for simplicity.

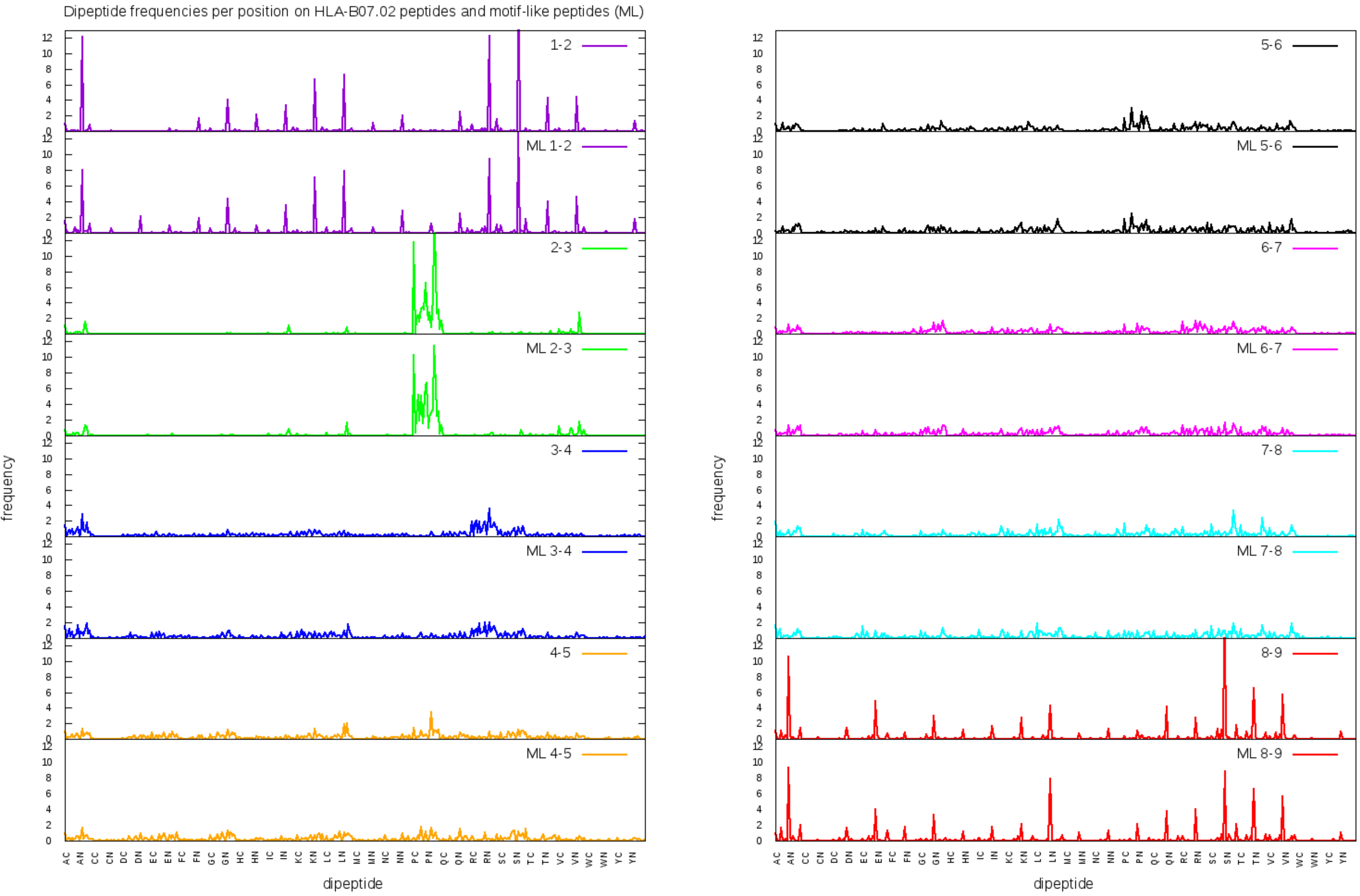

Figure. Dipeptide frequencies per pair-positions (1-2, 2-3, 3-4, 4-5, 5-6, 6-7, 7-8, 8-9) on 9-mer HLA-B*07:02 peptides and on 9-mer HLA-B*07:02 motif-like peptides (ML). 400 pairs of amino acids are possible to construct and therefore represented on x-axis. Just a few amino acids pairs are labeled for simplicity.

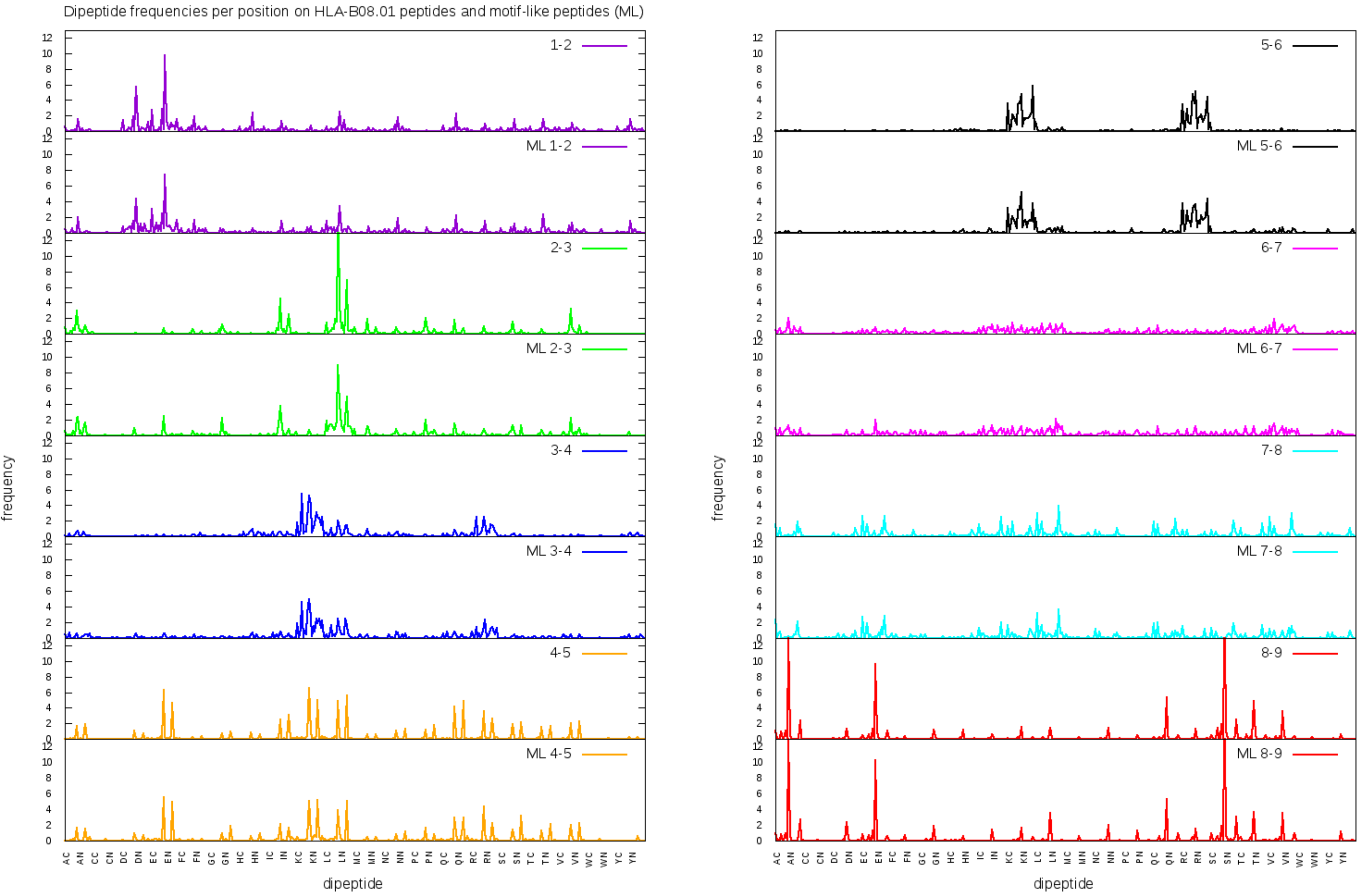

Figure. Dipeptide frequencies per pair-positions (1-2, 2-3, 3-4, 4-5, 5-6, 6-7, 7-8, 8-9) on 9-mer HLA-B*08:01 peptides and on 9-mer HLA-B*08:01 motif-like peptides (ML). 400 pairs of amino acids are possible to construct and therefore represented on x-axis. Just a few amino acids pairs are labeled for simplicity.
