# Supplementary material for: Analysis of Secondary Structure Biases in Naturally Presented HLA-I Ligands
Source: Front Immunol. 2019 Nov 22;10:2731. doi: 10.3389/fimmu.2019.02731 (PMC6883762; doi:10.3389/fimmu.2019.02731)
Supplement: Supplementary file 12 [file Data_Sheet_7.PDF]

Table SI. Comparison between HLA-I-MS peptides and motif-like PDB with representation on proteome for 2 different alleles: HLA-B*44:02 and HLA-C*07:02. Values for the fitness function *d* compare (I) HLA-I-MS (known binders) and HLA-I-MS-PDB (known binders with PDB match) (II) motif-like (non-binders, yet with similar PWM) and HLA-I-MS peptides. Average (AVG) of the amount of coil, helix, strand and respective standard deviation (STDEV) is present for each allele.

Allele		*d*	Coil		Helix		Strand	
			AVG	STDEV	AVG	STDEV	AVG	STDEV
HLA-B*44:02	HLA-I-MS peptides	1.66	0.32	0.02	0.47	0.03	0.21	0.02
	motif-like peptides	1.16	0.31	0.01	0.45	0.03	0.24	0.02
HLA-C*07:02	HLA-I-MS peptides	1.26	0.38	0.01	0.34	0.02	0.28	0.02
	motif-like peptides	1.39	0.40*	0.01	0.32*	0.02	0.28	0.01

STDEV is the standard deviation of the mean calculated considering 100 times 80% of the peptides randomly taken.

HLA-B*44:02 and HLA-C*07:02 once again show a decrease in the amount of helix for motif-like peptides. To sum up, 6 alleles out of 7 studied in the present manuscript show a decrease in helix in the motif-like peptides and 1 allele show an equivalent amount of helix between the peptides and motif-like peptides. These results once again support the fact that 9-mer HLA-I binding peptides prefer helical secondary structures in their proteins of origin, and that this preference is not driven solely by their amino acid composition.
