# Supplementary material for: Analysis of Secondary Structure Biases in Naturally Presented HLA-I Ligands
Source: Front Immunol. 2019 Nov 22;10:2731. doi: 10.3389/fimmu.2019.02731 (PMC6883762; doi:10.3389/fimmu.2019.02731)
Supplement: Supplementary file 8 [file Data_Sheet_3.PDF]

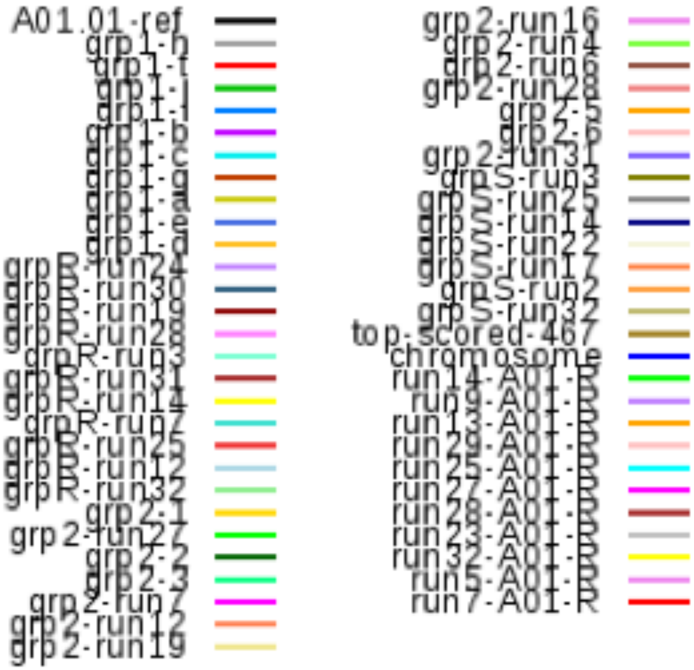

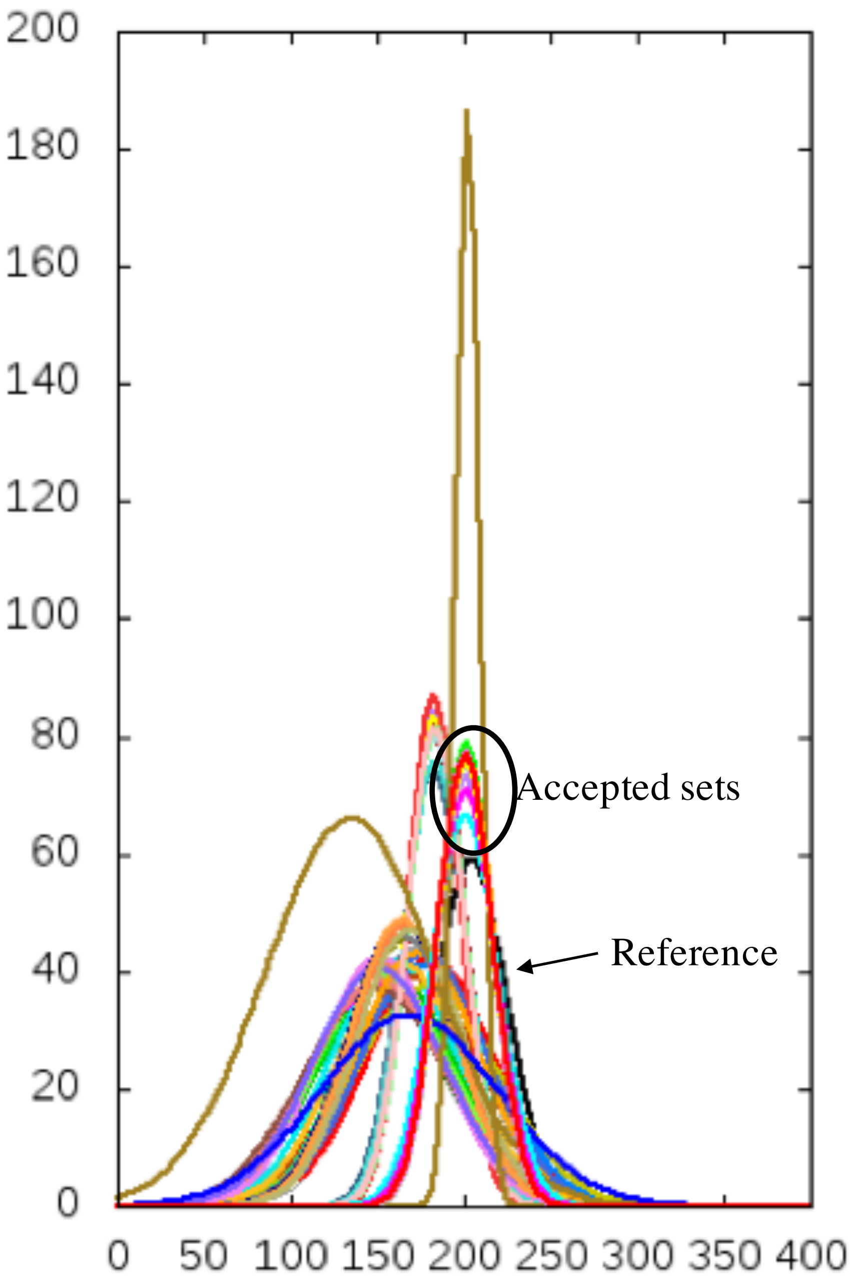

Figure. Probability distribution described by the fitting of a gaussian function to the histograms of the score *cf* of the peptides. Different converged motif-like sets for HLA-A01:01 are presented. Motif-like sets that present similar distribution of the *cf* conformity score as the reference set (gaussian function in black) are rounded by a black circle and were considered. Motif-like sets that present the same distribution of the *cf* conformity score as the reference set were discarded,
